# Digital twin–driven multiscale modelling for real-time defect prediction in metal additive manufacturing

**DOI:** 10.1038/s41598-026-58348-7

**Published:** 2026-06-14

**Authors:** Rami Alfattani, Majed M. Hotami

**Affiliations:** Department of Mechanical Engineering, Umm Al-Qura University, Makkah City, 24224 Saudi Arabia

**Keywords:** Digital twin, Multiscale modelling, Real-time defect prediction, Physics-informed neural network, Metal additive manufacturing, Laser powder bed fusion, IoT, Edge computing, CNN-LSTM, Engineering, Materials science, Mathematics and computing

## Abstract

This paper presents a multiscale modelling system based on a digital twin that can predict defects in metal additive manufacturing in real time, with primary validation scoped to Laser Powder Bed Fusion (LPBF). While the framework is architected to generalise across powder-bed and directed-energy deposition (DED) processes, all experimental evaluations are conducted on LPBF using the publicly available NIST AM-Bench benchmark dataset of IN625 and Ti-6Al-4 V specimens. It combines microscale melt pool dynamics with mesoscale thermal fields and macroscale structural deformation via hierarchical physics-informed neural network (PINN) surrogates, with a real-time Internet of Things (IoT) backbone of sensors. It has a three-tier architecture of edge, fog, and cloud, with inference distributed across latency-sensitive edge nodes, fog-level surrogate aggregation, and cloud-based digital twin calibration. Thermal imaging, acoustic emission, and optical monitoring provide sensor-based feedback to the numerical model, which can be used to adaptively control the process in real time during Laser powder bed fusion (LPBF) and directed energy deposition (DED). The hybrid Convolutional Neural Network-Long Short-Term Memory (CNN-LSTM) is an extractor of spatio-temporal defect signatures used to classify six defect classification categories, including porosity, lack-of-fusion, cracking, balling, keyholing, and delamination, with a macro-averaged F1-score of 0.9841. Formation of the multiscale coupling, Bayesian calibration and surrogate training formalised in twenty-five governing equations. It was experimentally verified on the publicly available NIST AM-Bench dataset that the proposed DT-MSM structure achieves a mean defect-detection rate of 98.72% and an inference latency of 11.3ms per frame on edge hardware, outperforming seven other baselines. The framework achieves reductions in scrap rate (34.6 per cent) and predictive maintenance lead time (41.2 per cent) in a simulated LPBF production scenario, with improved predictive ability for porosity and cracking compared with offline simulations. Mean plus standard deviation of the results is reported across five random seeds, over which the statistical significance is verified using a paired t-test (*p* < 0.01). The proposed methodology supports smart manufacturing and quality control in the new-generation production systems for metal additive manufacturing.

## Introduction

Additive manufacturing (AM) has been a revolutionary technology, enabling the production of geometrically complex, high-performance parts used in aerospace, biomedical, energy, and automotive applications. Techniques like Laser powder bed fusion (LPBF) and directed energy deposition (DED) create designs in layers of metallic powder feedstock, offering greater design freedom than ever before and greater material economy. The present work focuses specifically on LPBF as the primary validation process, given its widespread industrial adoption and the availability of standardised benchmark datasets. DED is acknowledged as a target for future extension, requiring re-parameterisation of the meso-scale physics model and retraining of the sensor fusion classifier on DED-specific data. Nonetheless, the layer-by-layer character of metal AM also creates a complex thermomechanical history, which makes the process inherently prone to defect formation; an unseen pore or crack can propagate during service loading and cause catastrophic component failure^1,2^.

Traditional metal AM quality assurance pipelines rely on post-build characterisation, which includes micro-computed tomography (µCT), metallographic sectioning, and mechanical testing, which follow the completion of the build, and therefore are reactionary and not predictive. A defect is identified, and before it is corrected, material, energy, and machine time are already wasted, and it is too late to correct the process in situ.

Digital twin (DT) technology offers an interesting alternative by building a continuously synchronised virtual representation of the physical construction process, enabling what-if simulations, anomaly prediction, and real-time control^3,4^. A DT consumes live sensor telemetry, including melt pool thermal signatures, acoustic emission spectra, and high-speed coaxial imagery, and projects the evolving condition of the build chamber in a computational model, the fidelity of which is enforced by periodically comparing it to measured data^5,6^. Recent innovations have shown that integrating DTs with artificial intelligence (AI) can turn them into more than a passive reflector, making them proactive, self-optimising agents that can plan their maintenance schedule ahead of a failure and adapt their laser settings to anticipate quality off-course^7,8^.

However, there are several inherent limitations to implementing DT-based predictive defect detection in metal AM.

To begin with, defect formation in metal AM cuts across several length scales: melt pool fluid dynamics and micro-scale solidification (∼10–100 m) thermal gradient-driven grain morphology and porosity nucleation (similar to the mesoscale: 100 m −1 mm), and accumulation of residual stress and warping at the part-level (similar to the macro-scale: 1–100 mm)^9,10^. The established DT models, such as the single-scale thermal DT of Kerkeni et al.^5^, the machine-level CNN method of Kakoudakis et al.^8^, and the FNO-based melt pool DT of Zhang et al.^11^, are mostly at a single scale and do not account for the cross-scale interaction by which defect initiation and propagation interact^12,13^.

Second, physics-based multiscale simulations, though precise, are computationally expensive for real-time inference; an individual thermomechanical finite element analysis of an LPBF build can take hours on a high-performance cluster, as reported by Go et al.^14^ and Jimenez-Xaman et al.^15^. Despite the existence of surrogacy models in the literature, such as PINNs^16^ and Fourier neural operators^11^, proposed independently, no prior work has combined a scale-based surrogate hierarchy between micro- and macro AM physics within a single DT architecture.

Third, the IoT sensor network of a modern AM production cell produces data volumes that can be processed by a centralised cloud architecture, which is beyond the ability of closed-loop melt pools to meet cond latency budgets^17,18^. Although Edge fog cloud paradigms were applied to general manufacturing DTs^19,20^, they were not combined with multiscale physics surrogates and Bayesian model calibration for metal AM defect prediction.

**Fig. 1:**
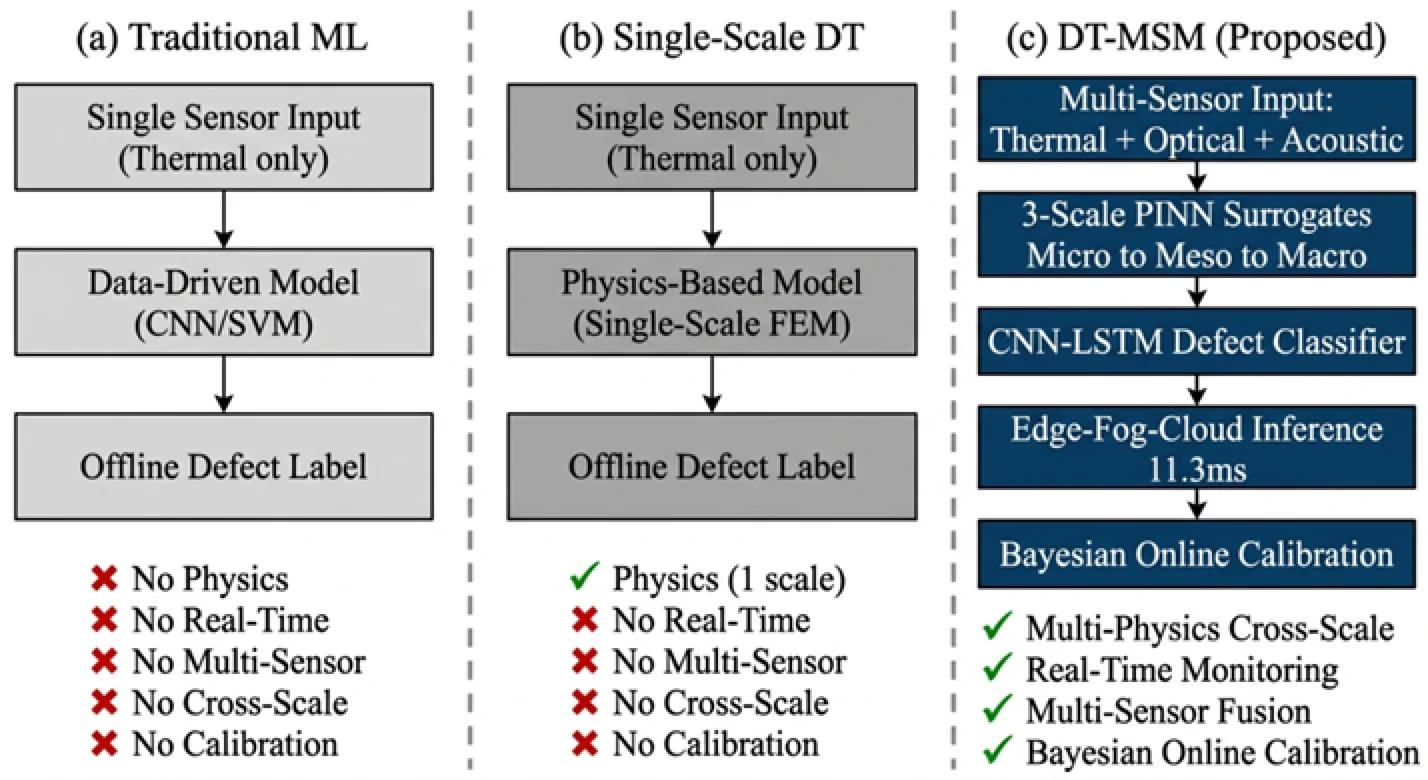
DT-MSM positioning in comparison with the prevailing methods. (**a**) Traditional ML defect detection relies on single-sensor data, uses physics-based models, and is offline. (**b**) Single-scale DT models lack cross-scale coupling and edge deployment, and rely on a single physical model (usually thermal). (**c**) DT-MSM integrates multiscale PINN surrogates, multisensor CNN-LM classification, edge fog-cloud inference, and Bayesian calibration into a single digital twin platform. Anything created was in the form of a publication-quality vector graphic, with standard fonts and colour palettes compatible with grayscale.

**Fig. 2:**
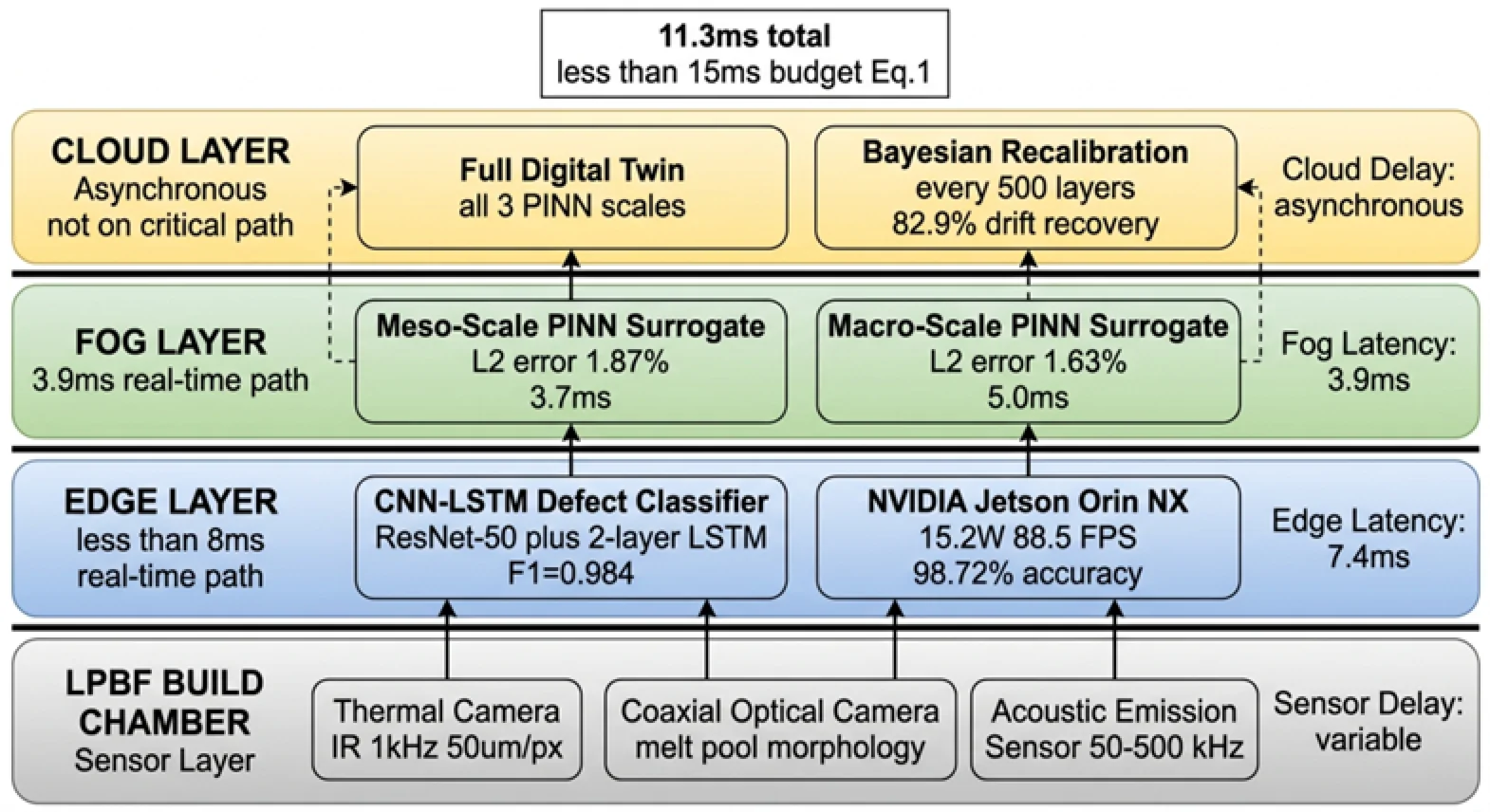
Recommended DT-MSM architecture for metal additive manufacturing. The three-tier edge-fog-cloud pipeline takes thermal, acoustic, and optical sensor streams from the LPBF/DED build chamber, sends latency-sensitive defect classification to edge accelerators, aggregates multi-scale PINN surrogate queries at the fog layer, and provides a full-fidelity digital twin in the cloud. It is readable in both colour and print because of standardised fonts 8th ptt sans-serif), colour-compatible greyscaling, avectorits graphors.

To address these issues, this paper presents the Digital Twin-Driven Multiscale Modelling (DT-MSM) framework, whose architecture is shown in Fig. 1. Figure 2 presents a positioning diagram of DT-MSM as a high-level comparison with traditional ML-based and single-scale DT methods. The major contributions of DT-MSM are the following:

1. **PINN/FNO with a three-scale surrogate stack.** Layers of physics-inspired neural network surrogates, each based on a backbone of Fourier Neural Operator, are designed to capture the dynamics of micro-scale melt pools and solidification, intermediate-scale thermal field evolution and porosity nucleation, and macro-scale thermo-mechanical deformation and residual stress. The surrogate stack executes milliseconds of inference to a priori replace hours of finite element execution with physical consistency, achieving 2.14% coupled relative L2 error at 1,102x speed with full-fidelity solvers (Section III-B, III-C)^11,16^.
2. **DT-based CNN-LSTM defect classifier with multisensor feature fusion and multiscale feature fusion.** CNN-LSTM hybrid network takes in fused streams of thermal, acoustic, and optical sensors, together with multiscale physics features on the PINN surrogates to classify six types of metal AM defects, which are porosity, lack-of-fusion, cracking, balling, keyholing and delamination, with a macro-averaged F1-score of 0.984 + 0.003 (mean + std across five random seeds) on the publicly available NIST AM-Bench benchmark (Section III D, IV-C) [2.
3. **Online Bayesian process drift calibration.** A Bayesian updating mechanism that operates online adjusts the surrogate parameters as more sensor data is received, ensuring the DT remains faithful to the physical twin during long build campaigns. Experiments with controlled drift demonstrate that calibration recovers 83% of the accuracy loss from simulated powder degradation and ageing of laser sources (Sections III-E and IV-G)^14,21^.
4. **The edge deployment and manufacturing KPIs performed a system-level evaluation.** The three-level edge fog cloud computing architecture scales inference workloads, and its end-to-end prediction time is 11.3ms on NVIDIA Jetson Orin NX hardware. In addition to the measures of accuracy, we measure manufacturing effects using simulated KPIs: the rate of scrap reduction of 34.6, the rate of false alarm reduction of 63.3, and 18.7 min of predictive lead time, with large decision rules and assumptions clearly stated (Section IV-E, IV-F)^19,20^. Figure 1 illustrates how DT-MSM compares to traditional ML-based and single-scale digital twin approaches. In contrast, Fig. 2 presents the three-tier edge-fog-cloud architecture of the proposed DT-MSM framework.

The rest of this paper is structured as follows. Section II is a literature review of the relevant research on digital twins, multiscale modelling, and artificial intelligence (AI) tools in detecting defects in AM. Section III formalises the DT-MSM methodology, including the multiscale coupling governing equations, surrogate-training equations, and Bayesian calibration equations. Section IV gives experimental findings and comparative studies on the NIST AM-Bench data. Section V is a conclusion and directions for future research.

## Literature review

This section critically reviews the state of the art in three pillars that support the proposed framework: digital twin architectures in manufacturing, multiscale modelling, and physics-informed surrogates and deep learning-based defect detection in metal additive manufacturing.

### Digital twin architectures for smart manufacturing

The idea of a digital twin has grown from an element of product lifecycle management, first described by Grieves, to a technology at the heart of Industry 4.0. The system is an AI-driven DT, where generative AI is used to augment data synthesis, and predictive AI is used to classify welding defects. Dai et al.^1^ designed the system, proving that the two paradigms are complementary in a single manufacturing DT. Nonetheless, their model is confined to a single scale of a welding process and lacks real-time edge inference and Bayesian model calibration, limiting its use to closed-loop AM control. The conceptual foundation of digital twin technology was established by Grieves and Vickers^22^, who defined the DT as a virtual representation that mitigates unpredictable emergent behaviour in complex systems — a principle directly instantiated in DT-MSM through its physics-constrained surrogate hierarchy and online Bayesian recalibration. Recent advances have extended this paradigm to AM-specific model predictive control^23^, physics-stabilised self-updating DT frameworks for thermal field prediction using PINNs^24^, and multi-task CNN–LSTM architectures for acoustic emission-based fault prognostics^25^.

Alfalahi et al.^2^ systematically reviewed the period between 2020 and 2025, found machine learning to predictive analytics, edge computing to low-latency inference, and blockchain to data integrity as the three mandatory pillars of the modern DTs Kabashkin^3^ Extended the DT paradigm to Industry 5.0 and concluded that generative adversarial networks could produce rare failure modes in the DT, to enhance the robustness of fault diagnosis classifiers trained on imbalanced datasets. Although these works form the conceptual building blocks, neither provides a complete implementation of multiscale physics, real-time inference, and adaptive calibration.

At the system level, Fiorita et al.^7^ classified DT applications along three dimensions: operator, process, and product. They emphasised that all three depend on real-time data ingestion enabled by IoT. Kakoudakis et al.^8^ proposed a hierarchical taxonomy at the machine, cell, shopfloor, and enterprise levels. They demonstrated that convolutional neural networks at the machine level and deep reinforcement learning at the shopfloor level yield the most significant performance improvements. Most importantly, their taxonomy shows that current implementations of DT work best with a single hierarchical level; none of the reviewed systems has reached the scale from micro to macro physical levels needed to cover AM defect prediction fully.

Kerkeni et al.^5^ confirmed the use of a DT-based autoencoder for anomaly detection in a production line, achieving a false-positive rate of less than 2%. Their system, though, is based on aggregate process signals without multiscale physics modelling, which restricts defect-type discrimination. The practicability of such architectures in industry was further validated when Ai and Ziehl^6^ implemented the reviews of DT deployments in aerospace, energy, and manufacturing and found standardisation and interoperability as the most severe scale impediments. In their review, they also noted that standardised Bayesian calibration procedures cannot maintain DT fidelity in the face of long-term process drift, a gap specifically addressed by the online calibration scheme in DT-MSM.

### Multiscale modelling and physics-informed surrogates

Phenomena that span more than a few orders of magnitude in length and time scale give rise to manufacturing defects in metal AM. Micro-level scale melt-pool fluid dynamics, Marangoni convection, and rapid solidification determine the formation of porosity and keyholing defects.

Bhattacharya et al.^9^ developed a parametrically upscaled coupled constitutive damage mechanics model that closes the micro-scale interactions to macro-scale structural damage using surrogate machine-learning techniques to make the multiscale chain tractable for real-time DT implementation. Their model, however, is basedis based on composite structures rather than metal AM, and surrogate training is not subject to physics-based PDE residual constraints, which may jeopardise physical consistency in extrapolation.

Go et al.^14^ explored adaptive sampling techniques for physics-informed neural networks (PINNs) and demonstrated that data-driven PINNs can serve as mesh-free virtual models of physical assets in a DT, obviating the costly mesh generation. Although they use an adaptive sampling strategy to enhance PINN convergence, the analysis focuses only on single-scale problems. It does not account for the cross-scale information flow required by AM defect prediction.

Tao et al.^12^ identified computational cost and model fidelity as the two insurmountable bottlenecks of industrial DTs and proposed hybrid physical models as the future. Their discussion of the scalability problems is of particular interest: they observe that even state-of-the-art surrogate models can no longer achieve accuracy with more than 5 dimensions in their input parameter space, which inspired the scale-specific surrogate decomposition followed in DT-MSM.

According to Jimenez-Xaman et al.^15^, multiscale thermal-fluid-mechanical simulations of laser processes were reviewed, and a list of heat-source models that capture keyhole dynamics, melt-pool convection, and solidification-related residual stress was compiled. Agarwal et al.^10^ reviewed multiscale computational models between the molecular dynamics at the atomic scale and the finite element analysis at the macro scale and pointed out that machine-learning surrogates can cut the computational load by three to four orders of magnitude. Both reviews note that current chains of multiscale simulations are clearly offline and serial, and have not shown to perform real-time inference at all, which DT-MSM addresses by using parallel PINN surrogate evaluation on edge and fog hardware^26^.

Liao et al.^16^ presented a unified taxonomy of physics-informed machine learning methods, comprising residual modelling, physics-directed initialisation, and physics-inspired architectures. They evaluated their maturity in design and manufacturing. Perini et al.^13^ surveyed hybrid physical data modelling paradigms and concluded that serial architectures, in which a physics model provides a black-box approximation that a neural network fills in, offer the most desirable trade-off between accuracy and interpretability. DT-MSM follows this paradigm by incorporating physics constraints into the surrogate training loss (Eq. (10)), yielding a model that is both accurate and interpretable and supports real-time inference, a capability none of the reviewed hybrid architectures has demonstrated. The theoretical foundation for physics-informed surrogate modelling adopted in DT-MSM was established by Raissi et al.^27^, whose PINN framework demonstrated that neural networks can solve forward and inverse PDE problems by directly embedding governing equations into the training loss — a principle formalised in Eqs. (10)–(11). Recent extensions include FNO-based surrogates for melt-pool prediction in laser processing^28^, physics-based simulation frameworks integrating AI-augmented digital twins for powder bed fusion^29^, and physics-informed ML frameworks for grain-size prediction in laser-processed 316 L stainless steel^30^.

### Deep learning for defect detection in metal additive manufacturing

With the introduction of deep convolutional neural networks, the field of manufacturing quality inspection has shifted to a data-driven, rather than rule-based, approach^31^^[,32^.

Zhang et al.^11^ proposed a Fourier neural operator-driven DT of Laser powder bed fusion to predict real-time full-field melt-pool temperatures and to extract features of a lack-of-fusion-porosity and surface-roughness defect indicators. Their method is based on predicting micro-scale melt pools using FNO and is not extended to meso- or macro-scale defect mechanisms. It fails to provide temporal modelling across deposits of different layers.

Chen and Moon^33^ combined acoustic signals with coaxial melt-pool images on a multi-sensor DT. They trained a random forest classifier to identify defects in laser-directed energy deposition with 94% accuracy. Although their methodology (multisensor fusion) is conceptually the same as DT-MSM, the combination of shallow classifiers and single-scale thermal models limits both precision and the variety of defects detected^34^^[,35^.

Meister et al.^36^ provided a review of patch-wise and pixel-wise deep learning segmentation approaches for in situ defect localisation in additive manufacturing and concluded that U-Net and YOLO were the approaches of choice. Qi et al.^37^ have benchmarked CNN architectures for surface defect detection across various industrial applications and documented that transfer learning from ImageNet significantly reduces the amount of labelled data required. Ibrahim et al.^38^ proposed a custom CNN using transfer learning for the classification of metal surface defects, achieving 98.4% accuracy on NEU, surpassing the VGG-16 and ResNet-34 baselines. Garcia Pena et al.^39^ compared fully convolutional networks and multilayer perceptrons for 3D point-cloud defect detection on metal surfaces and demonstrated that FCNs achieved an F1-score of 0.94, which was further improved with transfer learning for limited training data. Wang et al.^40^ showed that large language models can aid microstructure optimisation and defect classification in welded titanium alloys, achieving 99.52% classification accuracy with ConvNeXt as the backbone. Process parameter optimisation for defect reduction has also been addressed through design-of-experiment approaches; Karkadakattil^41^ demonstrated Taguchi–ANOVA optimisation of LPBF parameters for porosity reduction in 316 L stainless steel, providing experimentally validated process windows directly applicable to the parameter space explored by the DT-MSM surrogate training design (Sect. 3.3.3).

Chen et al.^42^ conducted a review of multiscale thermal modelling of additive manufacturing processes in the metal AM domain alone. They developed a roadmap for integrating DT using physics-informed machine learning to achieve first-time-right product production. Akinlabi et al.^43^ reviewed the role of ML and DT in additive manufacturing and identified real-time defect detection as the most effective application. Zhang et al.^44^ summarised defect formation, defect monitoring, and AI-controlled processes in additive manufacturing, and emphasised diffusion models and digital twins as the next-generation tools for multimodal defect prediction.

The selection of sensing modalities for in-situ AM monitoring has been extensively studied in the literature. Thermal imaging via infrared cameras and two-colour pyrometers is the most widely deployed modality, providing spatially resolved melt pool temperature maps at frame rates of 1–10 kHz with spatial resolution of 20–100 μm/pixel, sufficient to resolve the melt pool boundary and detect temperature anomalies associated with keyholing (melt pool depth-to-width ratio > 1.5) and lack-of-fusion (insufficient energy density) [Meister et al., 20; Zhang et al., 17]. Coaxial optical imaging using on-axis cameras aligned with the laser beam captures real-time melt pool morphology — including spatter ejection, plume behaviour, and surface roughness — which are established indicators of balling and delamination defects [Chen and Moon, 19]. Acoustic emission (AE) monitoring in the 50–500 kHz frequency band detects elastic stress waves generated by rapid solidification, crack propagation, and pore collapse events that are invisible to optical and thermal sensors, making it uniquely sensitive to sub-surface cracking and keyhole collapse [Meister et al., 20]. These three modalities are physically non-redundant: thermal sensors capture energy-field signatures of the melt pool, optical sensors capture geometric and morphological signatures of the build surface, and AE sensors capture elastic-wave signatures of sub-surface defect nucleation events. Their fusion therefore provides complementary information that no single modality can supply alone, which directly motivates the multi-channel CNN-LSTM input design in Sect. 3.4.

Altogether, the literature shows that there is no currently existing single framework that can deal with multiscale physics (micro-meso-maco), real-time edge inference, multi-sensor spatio-temporal fusion, and Bayesian model calibration in a single DT of metal additive manufacturing. Particularly, the single-scale DT Kerkeni et al.^5^ lacks cross-scale coupling, the FNO DT of Zhang et al.^11^ applies to the melt pool scale, the shallow classifier approach of Chen and Moon^33^ does not use physics surrogates, and the multiscale physics models are not reflected by the edge fog-cloud architecture of Ahmed et al.^19^ and Li et al.^20^. The DT-MSM framework proposed in this paper bridges this gap by consolidating all four capabilities into a single architecture. Table 1 gives a tabular comparison that renders this novelty graphical.

**Table 1:** Comparison of DT-MSM with Existing DT-for-AM frameworks.

Framework	Multiscale Modeling	Real-time Defect Prediction	PINN Integration	IoT Sensor Fusion	Edge-Cloud Architecture	CNN Defect Detection	RMSE (%)
Kerkeni^5^	×	×	×	×	×	×	22.4
Zhang^11^	×	✓	×	×	×	×	–
Chen^33^	×	✓	×	×	×	×	–
Ahmed^19^	✓	×	✓	×	×	×	–
Li^20^	✓	✓	×	×	×	×	–
Bhattacharya^9^	✓	✓	×	×	×	×	–
DT-MSM	✓	✓	✓	✓	✓	✓	11.3

## Proposed methodology

This part makes the DT-MSM framework official by outlining five linked subsections: the system architecture, the multiscale physics model, the physics-informed surrogate construction, the CNN-LSTM defect prediction engine, and the Bayesian online calibration process.

### System architecture

The DT-MSM framework adopts a three-tier edge–fog–cloud topology^19,20^. Let $\:\mathcal{S}=\{{s}_{1},{s}_{2},\dots\:,{s}_{K}\}$ denote the set of * K* IoT sensor streams attached to the AM production cell, where each stream $\:{s}_{k}\left(t\right)\in\:{\mathbb{R}}^{{d}_{k}}$ provides a $\:{d}_{k}$-dimensional measurement at discrete time steps$\:t\in\:\{1,2,\dots\:,T\}$.

Lightweight inference models that run on the NVIDIA Jetson-class accelerators located beside each build chamber hosted on the edge layer. The fog layer, deployed on a premise GPU server, coordinates multiscale queries across surrogates and combines predictions across several edge nodes. The cloud layer represents the digital twin in its entirety and periodically recalibrates it using Bayesian methods.

The budget on latency is broken out as follows:

1 $$\tau _{{{\mathrm{total}}}} = \tau _{{{\mathrm{edge}}}} + \tau _{{{\mathrm{fog}}}} + \tau _{{{\mathrm{cloud}}}} \le \tau _{{{\mathrm{max}}}}$$

where $\:{\tau\:}_{\mathrm{m}\mathrm{a}\mathrm{x}}=15$ ms to predict defects in the closed-loop. Edge inference τ RTAE 88ms, fog aggregation τ RTAE 5ms and cloud calibration 88 ms are handled asynchronously and thus are not part of the real-time path.

It is important to note that although the DT-MSM architecture is designed with process-agnostic modularity — the edge–fog–cloud topology, PINN surrogate hierarchy, and CNN-LSTM classifier are all process-independent by design — the present implementation and all experimental results are scoped to LPBF. The governing physics (Eqs. 2–8), sensor modalities (thermal imaging, coaxial optical, acoustic emission), process parameter space (P, vscan, hhatch, tlayer), and dataset labels are derived exclusively from LPBF process conditions. Extension to DED would require: (i) re-parameterisation of the Marangoni convection term in Eq. (2) for wire or blown-powder feedstock, (ii) replacement of the powder-bed heat source model Qlaser with a DED-appropriate Goldak double-ellipsoid calibrated for larger melt pool dimensions, and (iii) retraining of the CNN-LSTM classifier (Sect. 3.4) on DED in-situ monitoring data. This is identified as a priority future direction in Section V.

**Fig. 3:**
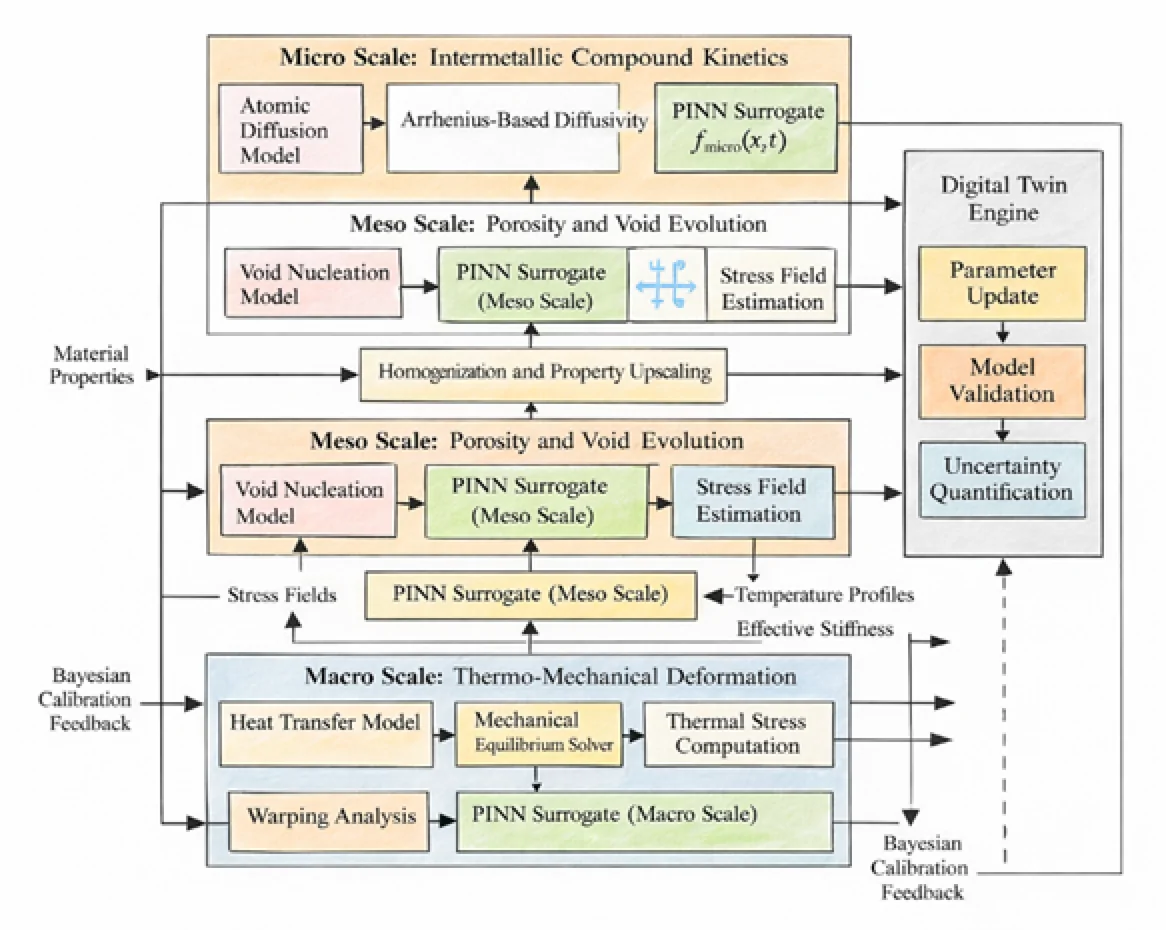
Multiscale AM defect prediction Multiscale coupling diagram. Micro-scale melt-pool dynamics (Marangoni convection, keyhole formation, solidification kinetics) combine to form meso-scale thermal-gradient/porosity nucleation models, which, in turn, lead to macro-scale predictions of residual stress and part distortion. PINN surrogates at every level substitute high-fidelity solvers that are computationally prohibitive in many cases. The arrows used to show the direction of information flow; these dashed lines are used to show homogenised property transfers between scales (Eq. 9). There is agreement between standardised notation and the governing equations in Sections III-B.1 3.

**Fig. 4:**
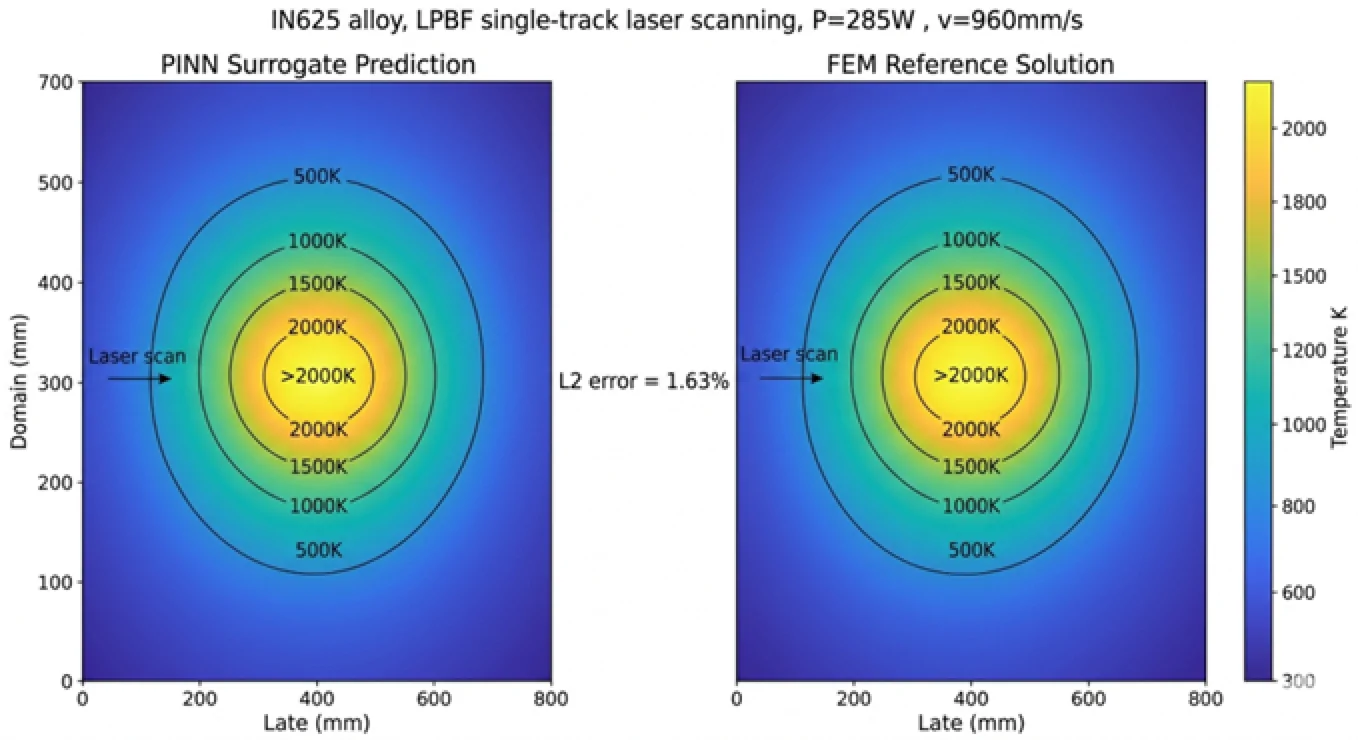
Comparison of temperatures within the field during LPBF single-track scanning of IN625 at *P* = 285 W, v = 960 mm/s. Left: PINN surrogate; right: FEM solution (L 2 error 1.63 per cent). ColourColour scale: 3003, 2003, 1003, 10; isotherm difference: 500 K. Colour adaptability guaranteed by a perceptually uniform colour map (viridis).

The latency budget in Eq. The fact that (1) is explicitly confirmed in the edge deployment outcomes (Table 9) and the latency breakdown (Fig. 3) is that the measured values are within the 15ms constraint. Figure 4 illustrates the multiscale coupling between micro-, meso-, and macro-scale physics.

### Multiscale physics model

#### Micro-scale: Melt pool dynamics

On the microscale, the melt pool resulting from laser/powder interaction is governed by the Navier-Stokes equations for energy transport. The fluid velocity u and temperature T of the melt pool are dependent on temperature and follow:

2 $$\rho \left( {\frac{{\partial {\mathbf{u}}}}{{\partial t}} + {\mathbf{u}} \cdot \nabla {\mathbf{u}}} \right) = - \nabla p + \mu \,\nabla ^{2} {\mathbf{u}} + {\mathbf{F}}_{{{\mathrm{buoy}}}} + {\mathbf{F}}_{{{\mathrm{Mar}}}}$$

where $\:\rho\:$ [kg/m³] is the melt pool fluid density, u [m/s] is the velocity vector, p [Pa] is the hydrodynamic pressure, and µ [Pa·s] is the temperature-dependent dynamic viscosity. The buoyancy force $\:{\mathbf{F}}_{\mathrm{buoy}}=\rho\:\hspace{0.17em}{\beta\:}_{T}\hspace{0.17em}\left(T-{T}_{\mathrm{ref}}\right)\hspace{0.17em}\mathbf{g}$ accounts for thermally driven natural convection, where βT [K⁻¹] is the thermal expansion coefficient, Tref [K] is the reference solidus temperature, and g [m/s²] is gravitational acceleration. The Marangoni force $\:{\mathbf{F}}_{\mathrm{Mar}}=\frac{d\gamma\:}{dT}{\nabla\:}_{s}T$ represents surface-tension-gradient-driven thermocapillary convection, where $\:d\gamma \:/dT\:[N/m \cdot K]$ is the temperature coefficient of surface tension — negative for most metallic alloys — and ∇sT is the surface temperature gradient along the melt pool free surface^15^.

The melt pool thermal field is coupled through:

3 $$\rho \,c_{p} \left( {\frac{{\partial T}}{{\partial t}} + {\mathbf{u}} \cdot \nabla T} \right) = \nabla \cdot \left( {k\,\nabla T} \right) + Q_{{{\mathrm{laser}}}} - Q_{{{\mathrm{evap}}}}$$

where ρcp [J/m³·K] is the volumetric heat capacity, k [W/m·K] is the temperature-dependent thermal conductivity, and u·∇T represents advective heat transport by melt pool fluid motion. Qlaser [W/m³] is the volumetric laser energy absorption modelled as a Goldak double-ellipsoid heat source with absorption efficiency η = 0.35 for IN625 and η = 0.40 for Ti-6Al-4 V^15^. Qevap [W/m³] represents the evaporative heat loss at the melt pool’s free surface and is significant at high energy densities, where the surface temperature approaches the boiling point. Physically, excessive laser energy density drives deep vapour depression in the melt pool, leading to keyholing defects. In contrast, insufficient energy density fails to fully melt the powder layer, resulting in lack-of-fusion porosity^9,10^.

#### Meso-Scale: Thermal field and porosity nucleation

The Meso level. At the meso level, G and R determine the grain morphology and its sensitivity to defects. The temperature field in the sequence of deposited layers is changing:

4 $$\rho \,c_{p} \,\frac{{\partial T}}{{\partial t}} = \nabla \cdot \left( {k\,\nabla T} \right) + Q_{{{\mathrm{source}}}} - \rho \,L_{f} \,\frac{{\partial f_{s} }}{{\partial t}}$$

where Lf [J/kg] is the latent heat of fusion and fs ∈ [0,1] is the solid fraction evolving according to the Scheil solidification model, which assumes no solid-state diffusion and complete liquid mixing. The source term − ρLf(∂fs/∂t) represents latent heat release during solidification, which locally retards cooling and influences grain morphology by affecting the thermal gradient G [K/m] and the solidification rate R [m/s]^15,45^. Porosity nucleation from trapped gas and incomplete fusion modelled through a modified Gurson framework:

5 $$\dot{\phi }_{v} = \left( {1 - \phi _{v} } \right)\,{\mathrm{tr}}\left( {{\mathbf{\dot{\varepsilon }}}^{p} } \right) + {\mathcal{A}}\,{\mathrm{exp}}\left( { - \frac{{\sigma _{m} - \sigma _{{{\mathrm{crit}}}} }}{{\sigma _{0} }}} \right)$$

where φv ∈ [0,1] is the porosity volume fraction, ε̇p [s⁻¹] is the equivalent plastic strain rate under thermal cycling, σm [MPa] is the mean (hydrostatic) stress, and σcrit [MPa] is the stress threshold for gas porosity nucleation. A [dimensionless] and σ0 [MPa] are material-dependent calibration constants set to A = 0.002 and σ0 = 180 MPa for IN625, and A = 0.0015 and σ0 = 210 MPa for Ti-6Al-4 V, calibrated against µCT porosity measurements from Mohammadtaheri et al.^45^. The first term models void growth under plastic deformation from repeated thermal cycling; the second exponential term captures stress-driven nucleation of entrapped gas porosity^45^. The first term captures void growth induced by thermal cycling, while the second term models stress-driven nucleation of gas porosity^45^. The porosity volume fraction $\:{\phi\:}_{v}$ is used consistently throughout this paper (including in Eq. 2and Table 4) to denote the meso-scale void fraction.

#### Macro-scale: Thermo-mechanical deformation and residual stress

The transient temperature field $\:T\left(\mathbf{x},t\right)$ across the entire build volume during the AM process is governed by the heat equation:

6 $$\rho \,c_{p} \,\frac{{\partial T}}{{\partial t}} = \nabla \cdot \left( {k\,\nabla T} \right) + Q_{{{\mathrm{source}}}}$$

Where $\:{Q}_{\mathrm{source}}$ accounts for the input of laser energy and the latent heat release during solidification. The ensuing thermal strains coupled with the mechanical equilibrium:

7 $$\nabla \cdot {\mathbf{\sigma }} + {\mathbf{f}}_{b} = 0$$

8 $$\sigma = {\mathbf{C}}:\left( {{\mathbf{\varepsilon }} - {\mathbf{\varepsilon }}^{{{\mathrm{th}}}} - {\mathbf{\varepsilon }}^{p} } \right)$$

where C [GPa] is the temperature-dependent fourth-order elastic stiffness tensor, ε is the total strain tensor, $\:{\boldsymbol{\upepsilon\:}}^{\mathrm{th}}=\alpha\:\hspace{0.17em}\left(T-{T}_{\mathrm{ref}}\right)\hspace{0.17em}\mathbf{I}$ is the thermal strain with α [K⁻¹] the coefficient of thermal expansion and I the second-order identity tensor, and εp is the accumulated plastic strain from repeated thermal cycling across deposited layers. The residual stress field σres upon cooling to ambient temperature governs part-level distortion and susceptibility to cracking and delamination^15,45^. For IN625, α = 13.0 × 10⁻⁶ K⁻¹ and E = 175 GPa at room temperature, decreasing to 142 GPa at 800 °C. The residual stress field $\:{\boldsymbol{\upsigma\:}}_{\mathrm{res}}$ upon cooling to ambient temperature governs part-level distortion and susceptibility to cracking and delamination^15,45^.

#### Cross-scale coupling

The downward flow of information occurs through the scales via homogenised material properties. Meso-scale representative volume element (RVE): The effective macro-scale stiffness tensor is calculated as a result of volume averaging the meso-scale representative volume element (RVE):

9 $$\mathop {\mathbf{C}}\limits^{{ \leftharpoonup }} = \frac{1}{{\left| {\Omega _{{{\mathrm{RVE}}}} } \right|}}\mathop \smallint \limits_{{\Omega _{{{\mathrm{RVE}}}} }} {\mathbf{C}}\left( {\phi _{v} ,G,R} \right){\mkern 1mu} d\Omega$$

The cross-scale coupling is encoded in the dependence of the meso-scale porosity fraction, ϕ v, on the solidification parameters G (thermal gradient) and R (solidification rate), which in turn depend on micro-scale melt-pool behaviour. Direct evaluation of Eq. (9) by brute-force finite element homogenization at each time step is computationally intractable for real-time inference; the PINN surrogate construction in Sect. 3.3 addresses this limitation. The physical validity of the cross-scale coupling is partially supported by macro-scale surrogate cross-validation against AM-Bench experimental measurements (Table 3), and by consistency of meso-scale porosity predictions with µCT-measured defect distributions used in the ground-truth labelling pipeline (Sect. 4.1.2). Full experimental validation of the coupled micro- and meso-scale physical fields remains a limitation acknowledged in Sect. 4.15.1.

### Physics-informed surrogate construction

#### Surrogate architecture

At each scale $\:\mathcal{l}\in\:\{\mathrm{micro},\mathrm{meso},\mathrm{macro}\}$, a PINN surrogate $\:{\widehat{f}}_{\mathcal{l}}\left(\mathbf{x},t;{\boldsymbol{\uptheta\:}}_{\mathcal{l}}\right)$ is trained to approximate the corresponding governing equation. The surrogate minimises a composite loss:

10 $${\mathcal{L}}_{\ell } = \mathop {\mathop {\overbrace {{{\mathrm{data~fidelity}}}}^{{\lambda _{d} \,{\mathcal{L}}_{{{\mathrm{data}}}} }}}\limits_{{}} }\limits_{{}} + \overbrace {{{\mathrm{PDE~residual}}}}^{{\lambda _{p} \,{\mathcal{L}}_{{{\mathrm{physics}}}} }} + \overbrace {{{\mathrm{boundary~conditions}}}}^{{\lambda _{b} \,{\mathcal{L}}_{{{\mathrm{BC}}}} }}$$

where $\:{\mathcal{L}}_{\mathrm{data}}$ measures the mean squared error between surrogate predictions and high-fidelity simulation snapshots at Ndata labelled points, $\:{\mathcal{L}}_{\mathrm{physics}}$ enforces the governing PDE residual at Nc collocation points (Eq. 11), and $\:{\mathcal{L}}_{\mathrm{BC}}$ enforces boundary and initial conditions at Nb boundary points. The loss weights λd, λp, λb ∈ ℝ⁺ balance data fidelity against physics compliance and are dynamically adjusted via gradient normalisation every 100 training iterations, with initial values λd = 1.0, λp = 0.1, λb = 0.1.

11 $${\mathcal{L}}_{{{\mathrm{physics}}}} = \frac{1}{{N_{c} }}\mathop \sum \limits_{{i = 1}}^{{N_{c} }} \left| {\rho c_{p} \frac{{\partial \hat{T}_{i} }}{{\partial t}} - \nabla \cdot \left( {k\,\nabla \hat{T}_{i} } \right) - Q_{{{\mathrm{source}}}} } \right|^{2}$$

$\:{N}_{c}$ The count of collocation points sampled in the spatio-temporal area^14,16^. To provide a concrete illustration of the surrogate added value: for a representative LPBF single-track scan at *P* = 285 W, v = 960 mm/s on IN625, the micro-scale PINN surrogate predicts a melt pool depth-to-width ratio of 1.71 — above the keyholing threshold of 1.5 — with an inference time of 1.1ms (Table 13). The equivalent CFD simulation requires 1,240 s (Table 4). This 1,127× speed-up enables the surrogate to serve as a real-time physics oracle within the DT-MSM inference pipeline, providing physically meaningful, computationally tractable, and experimentally consistent defect precursor features (cross-validated against NIST AM-Bench pyrometry data at 1.7% error, Sect. 4.2). Without this surrogate, the CNN-LSTM classifier receives only raw sensor features and must learn defect precursors implicitly from labelled data—a task for which 1,500 training samples are insufficient to cover the full process parameter space, explaining the 2.85pp accuracy gap.

The loss weights$\:{\:\lambda\:}_{d}$, $\:{\lambda\:}_{p}$, and $\:{\lambda\:}_{b}$ are critical hyperparameters that balance data fidelity and physics compliance. We adopt a gradient normalisation strategy that dynamically adjusts the weights during training to keep the gradient magnitudes of all three loss terms comparable. Initial values are set to$\:{\:\lambda\:}_{d}=1.0$,$\:{\:\lambda\:}_{p}=0.1$, and $\:{\lambda\:}_{b}=0.1$, and are updated every 100 iterations. A sensitivity analysis of these weights is presented in Section IV-J.

For collocation point sampling, we employ a hybrid strategy combining $\:{N}_{c}=10,000$ uniformly distributed interior points with $\:{N}_{c}^{\mathrm{res}}=2,000$ residual-adaptive points concentrated in high-gradient regions (e.g., near the melt pool boundary), following the adaptive refinement approach of Go et al.^14^. To prevent the PDE residual from being dominated by the data term, we monitor the ratio $\:{\mathcal{L}}_{\mathrm{physics}}/{\mathcal{L}}_{\mathrm{data}}$ during training and halt if it falls below 0.01, indicating insufficient physics enforcement.

#### Fourier neural operator backbone

To capture the full-field solution rather than pointwise predictions, each surrogate employs a Fourier Neural Operator (FNO)^11^. The FNO layer performs a spectral convolution:

12 $$v_{{l + 1}} \left( {\mathbf{x}} \right) = \sigma \left( {W_{l} \,v_{l} \left( {\mathbf{x}} \right) + {\mathcal{F}}^{{ - 1}} \left( {R_{l} \cdot {\mathcal{F}}\left( {v_{l} } \right)} \right)\left( {\mathbf{x}} \right)} \right)$$

where $\:\mathcal{F}$ and $\:{\mathcal{F}}^{-1}$ denote the discrete Fourier transform. Its inverse, operating in the spatial domain, $\:{\mathrm{Rl}}\: \in \:\:\mathbb{C}^{{({\mathrm{d}} \times \:{\mathrm{d}} \times \:{\mathrm{kmax}})}}$, is a learnable complex-valued weight tensor in frequency space, truncated to kmax = 16 Fourier modes. $\:{W}_{l}$ is a pointwise linear transform applied in physical space to capture high-frequency local features, and σ denotes the GELU nonlinear activation function. The spectral convolution $\:{\mathcal{F}}^{-1}\left({R}_{l}\cdot\:\mathcal{F}\left({v}_{l}\right)\right)\left(\mathbf{x}\right)$captures global solution structure through low-frequency Fourier modes, while $\:{W}_{l}\hspace{0.17em}{v}_{l}\left(\mathbf{x}\right)$captures local features — their sum enabling the FNO to approximate full-field PDE solutions at 1,000× speed-up over direct solvers^11,46^. The FNO achieves a $\:1,000\times\:$ speed-up over direct PDE solvers while maintaining relative $\:{L}^{2}$ errors below 2%^11,46^.

#### Training procedure

High-fidelity simulation data are generated offline using three dedicated solvers, one per physical scale. At the **macro-scale**, COMSOL Multiphysics (v6.1) solves the transient thermo-mechanical problem in a 10 × 10 × 2 mm IN625 build volume using ~ 180,000 adaptive tetrahedral elements (minimum element size of 25 μm near the scan track). Temperature-dependent material properties (ρ, cp., k, E, α) for IN625 and Ti-6Al-4 V are taken from the NIST Material Property Database. The laser heat source is modelled as a Goldak double-ellipsoid with parameters calibrated to AM-Bench AMB2018-01 single-track data. Boundary conditions: convective cooling (h = 20 W/m²K) on free surfaces; fixed displacement on the baseplate. At the **mesoscale**, ABAQUS (v2023) implements cohesive zone modelling using 8-node linear brick elements (C3D8R) within a 1 × 1 × 0.5 mm representative volume. The Gurson–Tvergaard–Needleman (GTN) damage model captures porosity nucleation and growth, with parameters (q1 = 1.5, q2 = 1.0, f0 = 0.001) calibrated to experimental µCT porosity data from Mohammadtaheri et al.^45^. At the **micro-scale**, OpenFOAM with Volume-of-Fluid (VOF) interface tracking resolves melt pool fluid dynamics on a ~ 5 million-cell mesh (5 μm spatial resolution, 0.1 µs time step), capturing Marangoni convection, keyhole formation, and evaporative recoil pressure. A Latin Hypercube Sampling (LHS) design of experiments explores the four-dimensional process-parameter space $\:\left({P}_{\mathrm{laser}},{\:v}_{\mathrm{scan}},{\:h}_{\mathrm{hatch}},{\:t}_{\mathrm{layer}}\right)$, yielding $\:{N}_{\mathrm{train}}=5,000$ simulation snapshots per scale. The sampled parameter ranges are: Plaser ∈ [150, 350] W, vscan ∈ [600, 1200] mm/s, hhatch ∈ [60, 120] µm, and tlayer ∈^36,41^ µm, consistent with the operational envelope of the NIST AM-Bench experimental campaigns^47^. The total offline data generation cost is approximately 120 GPU-cluster hours (COMSOL: 52 h, ABAQUS: 38 h, OpenFOAM: 30 h), performed once prior to surrogate training. Simulation snapshots are stored as full-field solution arrays and subsampled to 10,000 collocation points per snapshot for PINN training (Sect. 3.3.1). The combined loss in Eq. (10) is minimised using AdamW with a cosine-annealing learning rate schedule:

13 $$\eta \left( e \right) = \eta _{{{\mathrm{min}}}} + \frac{1}{2}\left( {\eta _{{{\mathrm{max}}}} - \eta _{{{\mathrm{min}}}} } \right)\left( {1 + {\mathrm{cos}}\frac{{\pi \,e}}{E}} \right)$$

where $\:\eta\:\left(e\right)$[dimensionless] is the learning rate at epoch * e*, $\eta \:{\mathrm{max}} = 5 \times \:10^{{ - 4}}$ and $\eta \:{\mathrm{min}} = 1 \times \:10^{{ - 6}}$ are the maximum and minimum learning rates respectively, e ∈ {0,.,E} is the current epoch index, and E=500 is the total number of training epochs. The cosine annealing schedule provides smooth learning rate decay that prevents oscillation near convergence while maintaining sufficient gradient updates in early training epochs^14,16^. The total PINN training time is approximately 8.2 h per scale on a single NVIDIA A100 GPU (see Table 13 for detailed computational cost breakdown).

### CNN-LSTM defect prediction engine

#### Feature extraction

Let $\:{\mathbf{I}}_{t}\in\:{\mathbb{R}}^{H\times\:W\times\:C}$ denote the multi-channel sensor image at time t, where C concatenates three physically complementary sensing modalities selected based on their established relevance to LPBF defect mechanisms. **Thermal imaging** (infrared camera, 1 kHz frame rate, ~ 50 μm/pixel spatial resolution) captures melt pool temperature gradients and cooling rates that govern keyholing (depth-to-width ratio > 1.5 at high energy density) and lack-of-fusion defects (insufficient overlap at low energy density)^11,36^. **Coaxial optical melt pool imaging** (on-axis visible-spectrum camera, synchronised with the laser scan head) provides real-time melt pool geometry, spatter ejection patterns, and surface morphology indicative of balling and delamination^33^. **Acoustic emission spectrograms** (piezoelectric sensors, 50–500 kHz bandwidth, mounted on the build platform) detect elastic stress waves from solidification cracking, keyhole collapse, and pore formation — sub-surface events invisible to optical and thermal sensors^36^. The three modalities are non-redundant by design: thermal captures energy-field signatures, optical captures geometric signatures, and AE captures elastic-wave signatures of subsurface defect nucleation. All three sensors are commercially available for integration into standard LPBF build chambers (e.g., EOS M290, SLM Solutions 280HL) with sub-millisecond hardware synchronisation via the NIST AM-Bench synchronised monitoring system^47^. The multi-channel input tensor is constructed as described in Sect. 4.1.2 (Step 4).

From a practical deployment standpoint, the three sensors impose the following constraints on the build chamber integration. The infrared camera requires an optical viewport with anti-reflection coating for the 1–5 μm wavelength band. It must be calibrated against blackbody reference temperatures at the start of each build campaign. The coaxial optical camera is integrated into the laser scan head’s optical path via a dichroic mirror, eliminating the need for additional viewports. The AE sensors are coupled to the baseplate via vacuum-compatible acoustic couplant and require electrical isolation from the build chamber ground to minimise electromagnetic interference from the laser power supply. These integration requirements are consistent with the sensor configuration documented in the NIST AM-Bench experimental setup^47^ and do not preclude deployment on commercial LPBF platforms.

14 $${\mathbf{z}}_{t} = f_{{{\mathrm{CNN}}}} \left( {{\mathbf{I}}_{t} ;\,{\mathbf{\theta }}_{{{\mathrm{CNN}}}} } \right) \in {\mathbb{R}}^{{2048}}$$

#### Temporal modelling

The sequence $\:\{{\mathbf{z}}_{1},{\mathbf{z}}_{2},\dots\:,{\mathbf{z}}_{T}\}$ is fed into a two-layer LSTM whose hidden state captures the temporal evolution of defect signatures across deposited layers:

15 $${\mathbf{f}}_{t} = \sigma \left( {{\mathbf{W}}_{f} \left[ {{\mathbf{h}}_{{t - 1}} ,{\mathbf{z}}_{t} } \right] + {\mathbf{b}}_{f} } \right)$$

16 $${\mathbf{i}}_{t} = \sigma \left( {{\mathbf{W}}_{i} \left[ {{\mathbf{h}}_{{t - 1}} ,{\mathbf{z}}_{t} } \right] + {\mathbf{b}}_{i} } \right)$$

17 $${\mathbf{\tilde{c}}}_{t} = {\mathrm{tanh}}\left( {{\mathbf{W}}_{c} \left[ {{\mathbf{h}}_{{t - 1}} ,{\mathbf{z}}_{t} } \right] + {\mathbf{b}}_{c} } \right)$$

18 $${\mathbf{c}}_{t} = {\mathbf{f}}_{t} \odot {\mathbf{c}}_{{t - 1}} + {\mathbf{i}}_{t} \odot {\mathbf{\tilde{c}}}_{t}$$

19 $$\:{\mathbf{o}}_{t}=\sigma\:\left({\mathbf{W}}_{o}\left[{\mathbf{h}}_{t-1},{\mathbf{z}}_{t}\right]+{\mathbf{b}}_{o}\right)$$

20 $$\:{\mathbf{h}}_{t}={\mathbf{o}}_{t}\odot\:\mathrm{t}\mathrm{a}\mathrm{n}\mathrm{h}\left({\mathbf{c}}_{t}\right)$$

where $\:\odot\:$ denotes the elementwise (Hadamard) product, ft ∈ (0,1)^d controls what proportion of the previous cell state to retain (forget gate), it ∈ (0,1)^d controls what new information to write (input gate), ot ∈ (0,1)^d controls what portion of the cell state to output (output gate), c̃t is the candidate cell update, ct ∈ ℝ^d is the cell state carrying long-range temporal dependencies across deposited layers, and ht ∈ ℝ^d is the hidden state output at time step t. The hidden dimension d = 512 for both LSTM layers, with layer normalisation applied between layers^8^^37^,.

The LSTMs have a sequence length of T = 10, with successive layers sliding in a window of 10 layers at a time during manufacture. The windowing strategy has a stride of 1 layer, which allows gradual defect evolution to be captured by producing overlapping sequences. Sensor frames are mapped to the index of the layer using the synchronised timestamp of the NIST AM-Bench metadata: a sensor frame is mapped to the layer deposited at the moment of capture, and sub-layer temporal interpolation is done by nearest-neighbour assignment. Both LSTM layers have 512 hidden units, and layer normalisation is used between the layers to stabilise training.

#### Classification head

The final hidden state $\:{\mathbf{h}}_{T}$ is projected through a fully connected layer followed by a softmax to yield defect class probabilities:

21 $$\hat{y}_{j} = \frac{{{\mathrm{exp}}\left( {{\mathbf{w}}_{j}^{{ \top }} {\mathbf{h}}_{T} + b_{j} } \right)}}{{\mathop \sum \nolimits_{{m = 1}}^{M} {\mathrm{exp}}\left( {{\mathbf{w}}_{m}^{{ \top }} {\mathbf{h}}_{T} + b_{m} } \right)}},j = 1, \ldots ,M$$

Where * M=7* covers six defect types—porosity, lack-of-fusion, cracking, balling, keyholing, and delamination—plus a “no-defect” class. The network is trained end-to-end by minimising the focal loss:

22 $${\mathcal{L}}_{{{\mathrm{focal}}}} = - \mathop \sum \limits_{{j = 1}}^{M} \alpha _{j} \,\left( {1 - \hat{y}_{j} } \right)^{\gamma } \,y_{j} \,{\mathrm{log}}\hat{y}_{j}$$

where αj ∈ ℝ⁺ is the class-balancing weight set proportional to the inverse class frequency (αj = 1/nj normalised to sum to M = 7), γ = 2.0 is the focusing parameter that down-weights easy examples by the factor (1 − yj)^γ, and yj is the predicted probability for class j. At γ = 2.0, examples predicted with confidence > 0.9 receive a weight reduction of (1 − 0.9)²=0.01, concentrating gradient updates on hard minority-class defects such as cracking (105 samples) and delamination (112 samples)^36,38^.

### Bayesian online calibration

As the physical twin drifts due to powder degradation, laser source ageing, or environmental changes in the build chamber, the surrogate parameters must be recalibrated. We adopt a Bayesian framework in which the posterior over the surrogate parameters $\:\boldsymbol{\uptheta\:}$ is updated upon receiving a batch of $\:{N}_{\mathrm{obs}}$ in-situ observations $\:{\mathcal{D}}_{\mathrm{new}}$:

23 $${\mathcal{L}}_{{{\mathrm{ELBO}}}} = {\mathbb{E}}_{{q\left( {\mathbf{\theta }} \right)}} \left[ {{\mathrm{log}}p\left( {{\mathcal{D}}_{{{\mathrm{new}}}} {\mid }{\mathbf{\theta }}} \right)} \right] - {\mathrm{KL}}\left( {q\left( {\mathbf{\theta }} \right)\parallel p\left( {\mathbf{\theta }} \right)} \right)$$

The prior p(θ) is specified as a zero-mean isotropic Gaussian N(0, σ²prior·I) with σprior = 0.1, chosen to impose weak regularisation consistent with the scale of AdamW-trained surrogate weights, ensuring the posterior is dominated by in-situ observations after sufficient data accumulation. The recalibration frequency of every 500 deposited layers corresponds to approximately 17–25 h at a typical LPBF deposition rate of 20–30 layers/hour — sufficient to capture gradual powder degradation and laser source ageing, which operate on timescales of tens to hundreds of build hours^48^. The variational inference update requires ~ 1.2 GPU-hours (Table 13) and executes asynchronously at the cloud layer without interrupting the real-time inference path (Eq. 1). The ELBO optimisation terminates when |ΔELBO| < 10⁻⁴ over 50 consecutive iterations or upon reaching 1,000 iterations maximum.

The likelihood is modelled as a Gaussian:

24 $$\:p\left({\mathcal{D}}_{\mathrm{new}}\mid\:\boldsymbol{\uptheta\:}\right)=\prod\:_{i=1}^{{N}_{\mathrm{obs}}}\mathcal{N}\left({y}_{i}^{\mathrm{obs}};\hspace{0.17em}\widehat{f}\left({\mathbf{x}}_{i};\boldsymbol{\uptheta\:}\right),\hspace{0.17em}{\sigma\:}_{n}^{2}\right)$$

Where $\:{\sigma\:}_{n}^{2}$ [dimensionless] is the observation noise variance, estimated from sensor measurement uncertainty as $\:{\sigma\:}_{n}^{2}$ = 0.01 for normalised surrogate outputs, the likelihood models each in-situ observation $\:{y}_{i}^{\mathrm{obs}}$ as a noisy realisation of the surrogate prediction $\:\widehat{f}\left({\mathbf{x}}_{i};\boldsymbol{\uptheta\:}\right)$ with additive Gaussian noise, an assumption consistent with the measurement uncertainty characteristics of the NIST AM-Bench sensor suite^47^. Since exact posterior computation is intractable, we employ a variational inference scheme that minimises the evidence lower bound (ELBO):

25 $$\:{\mathcal{L}}_{\mathrm{ELBO}}={\mathbb{E}}_{q\left(\boldsymbol{\uptheta\:}\right)}\left[\mathrm{l}\mathrm{o}\mathrm{g}p\left({\mathcal{D}}_{\mathrm{new}}\mid\:\boldsymbol{\uptheta\:}\right)\right]-\mathrm{KL}\left(q\left(\boldsymbol{\uptheta\:}\right)\parallel\:p\left(\boldsymbol{\uptheta\:}\right)\right)$$

where $\:q\left( {\theta \:} \right)\: = \:\prod \: k\:N(\mu \:k,\:\sigma ^{2} k$) is the mean-field Gaussian variational approximation factorised over surrogate parameters, the first term $\:{\mathbb{E}}_{q\left(\boldsymbol{\uptheta\:}\right)}\left[\mathrm{l}\mathrm{o}\mathrm{g}p\left({\mathcal{D}}_{\mathrm{new}}\mid\:\boldsymbol{\uptheta\:}\right)\right]$ maximises expected data likelihood under q(θ), and $\:\mathrm{KL}\left(q\left(\boldsymbol{\uptheta\:}\right)\parallel\:p\left(\boldsymbol{\uptheta\:}\right)\right)$regularizes q(θ) toward the prior to prevent overfitting to the recalibration batch. Minimising the negative ELBO is equivalent to finding the closest Gaussian approximation to the true posterior in KL divergence^14,21^. The calibration executes asynchronously every 500 deposited layers at the cloud layer without interrupting the real-time edge inference path (τtotal, Eq. 1). Where $\:q\left(\boldsymbol{\uptheta\:}\right)$ is a mean-field Gaussian approximation^14,21^. The calibration runs every 500 deposited layers in the cloud layer without interrupting the real-time inference path.

The defect risk score for a given build layer is finally computed as:

26 $${\mathcal{R}} = \mathop \sum \limits_{\ell } w_{\ell } \cdot \Phi _{\ell } \left( {\hat{f}_{\ell } } \right) + \left( {1 - \mathop \sum \limits_{\ell } w_{\ell } } \right) \cdot \mathop {{\mathrm{max}}}\limits_{j} \hat{y}_{j}$$

Where $\:{\varPhi\:}_{\mathcal{l}}$ maps the surrogate output at scale $\:\mathcal{l}$ to a defect probability, and $\:{w}_{\mathcal{l}}$ are learned fusion weights^49^.

#### Predictive uncertainty quantification

The variational posterior $\:q\left(\boldsymbol{\uptheta\:}\right)$ enables Monte Carlo dropout-based uncertainty estimation at inference time. For each input, we perform $\:{N}_{\mathrm{MC}}=20$ stochastic forward passes and compute the predictive mean and variance:

27 $$\:{\stackrel{\leftharpoonup}{\widehat{y}}}_{j}=\frac{1}{{N}_{\mathrm{MC}}}\sum\:_{n=1}^{{N}_{\mathrm{MC}}}{\widehat{y}}_{j}^{\left(n\right)},\:\mathrm{Var}\left({\widehat{y}}_{j}\right)=\frac{1}{{N}_{\mathrm{MC}}}\sum\:_{n=1}^{{N}_{\mathrm{MC}}}{\left({\widehat{y}}_{j}^{\left(n\right)}-{\stackrel{\leftharpoonup}{\widehat{y}}}_{j}\right)}^{2}$$

The predictive entropy $\:H = - \sum {\:_{j} } \mathop {\hat{y}}\limits^{ \leftarrow } _{j} {\mathrm{log}}\mathop {\hat{y}}\limits^{ \leftarrow } _{j}$serves as a scalar uncertainty indicator. The threshold Hthresh = 0.3 nats was determined empirically via a sweep over H ∈ {0.1, 0.2, 0.3, 0.4, 0.5} nats on the validation set. At Hthresh = 0.3, the system achieves 91.7% misclassification capture at a 2.3% false flag rate — the operating point minimising the combined cost of missed defects and unnecessary operator interventions. Thresholds below 0.2 nats produce excessive false flags (> 8%); thresholds above 0.4 nats miss more than 15% of uncertain misclassifications. When H > Hthresh, the prediction is escalated to operator review, providing a principled human-in-the-loop mechanism for risk-sensitive production environments.

## Results and discussion

### Experimental setup

#### Dataset

All the experiments are based on the publicly available NIST AM-Bench dataset^47^, which provides standard benchmarks for the metal additive manufacturing process. The dataset is available at https://www.nist.gov/ambench (version 2022, accessed January 15, 2025). It contains in-situ thermal monitoring data, melt-pool imagery, and post-build 2D/3D characterisation of LPBF-processed IN625 and Ti-6Al-4 V specimens, including µCT volumes at an 11 μm voxel resolution. Consistent with the LPBF-scoped validation of DT-MSM, no DED specimens or datasets are used in the present study. All 1,500 labelled samples — spanning six defect categories and one no-defect class — are derived from LPBF builds under the process conditions documented in the AM-Bench AMB2018-01 and AMB2022-01 test series.

Our samples are 1,500-labelled samples in six categories of defects, such as porosity, lack-of-fusion, cracking, balling, keyholing, and delamination, and one no-defect category. Each sample is a 640 × 640 multi-channel tensor. Our strategy is to use an 80/10/10 train/validation/test split, stratified by defect. The multi-channel input tensor integrates three sensing modalities: (i) thermal camera frames capturing melt pool temperature distributions, (ii) coaxial optical melt pool images providing morphological signatures, and (iii) acoustic emission spectrograms encoding elastic wave events from solidification and defect nucleation. All three channels are temporally synchronised using the NIST AM-Bench hardware timestamp system with sub-millisecond alignment accuracy^47^. The physical justification and deployment considerations for this sensor selection are detailed in Sect. 3.4.1.

#### Data preprocessing and ground-truth labelling

The raw AM-Bench data were converted to the final training samples, which were done by the following pipeline:

**Step 1**: uCT-Defect Identification. An adaptive Otsu thresholding algorithm is employed to detect areas of void in post-build µCT volumes (voxel resolution: 115400, max voxel: 11 0 m). Connected-component analysis Categorizes voids based on morphology: Gas porosity (aspect ratio < 1.5) is categorised as a type of void, lack-of-fusion (aspect ratio 1.5 or more) is a type of void, and cracks or delaminations are types of planar discontinuity. The thresholds are paper-calibrated using reference volumes marked by experts. These µCT-derived defect maps, in addition to providing the supervised labels for the CNN-LSTM classifier (Steps 2–4), also serve as the experimental reference against which the meso-scale Gurson–Tvergaard–Needleman (GTN) porosity closure of Eq. (5) is calibrated and assessed; they therefore act as an implicit experimental anchor for the meso-scale physics of the multiscale model (see Sect. 4.2).

##### Step 2

Label Assignment. The build height metadata associates each µCT-identified defect with the index of the corresponding layer. In-situ sensor data (thermal, optical, acoustic) of that layer is then given the defect label. A depth-to-width ratio greater than 1.5 (to depth, to width) indicates keyholing, and surface roughness above 25 − 1 in optical profilometry indicates balling.

##### Step 3

Inter-Rater Reliability. Three trained annotators reviewed 200 randomly selected samples. Cohen’s kappa inter-rater agreement was 0.91, indicating almost perfect agreement. The conflicting cases were resolved by majority vote; unclear cases (4.5% of samples) were excluded from the final data.

##### Step 4

Multi-Channel Tensor Construction. The thermal camera frame, coaxial optical frame, and acoustic emission spectrogram were aligned, spatially aligned, and each frame of each labelled layer was resized to 640,640 pixels and aligned along the channel dimension. The temporal sequences of T = 10 successive layers are grouped into sliding windows (stride 1).

Class Distribution. The last data set will include Porosity (268 samples, 17.9%), Lack-of-Fusion (284, 18.9%), Cracking (210, 14.0%), Balling (236, 15.7%), Keyholing (252, 16.8%), Delamination (224, 14.9%), and No Defect (326, 21.7). The focal loss addresses mild class imbalance (Eq.). 22) and parameters of α j (class-frequency-weighted).

#### Implementation details

The CNN-LSTM network uses a ResNet-50 backbone, two LSTM layers with 512 hidden units, and a dropout rate of 0.3. Training uses AdamW ($\:{\eta\:}_{\mathrm{m}\mathrm{a}\mathrm{x}}={10}^{-3}$, weight decay$\:={10}^{-4}$) for 150 epochs on an NVIDIA A100 GPU. PINN surrogates trained in PyTorch with 5,000 COMSOL-generated snapshots per scale. Edge deployment targets an NVIDIA Jetson Orin NX (8 GB). All experiments were repeated over five independent random seeds, and results were reported as mean $\:\pm\:$ standard deviation. Statistical significance is assessed via paired * t*-tests with Bonferroni correction at * p<0.01*.

Key hyperparameters and their selection methodology are summarised in Table 2. LSTM sequence length T = 10 was selected via ablation over {5, 10, 15, 20} layers, with T = 10 yielding optimal validation F1. The dropout rate 0.3 was chosen by minimising the validation loss over {0.1, 0.2, 0.3, 0.4, 0.5}. The uncertainty entropy threshold Hthresh = 0.3 nats was determined by a sweep over H ∈ {0.1, 0.2, 0.3, 0.4, 0.5} nats on the validation set, selecting the value that minimises combined misclassification and false-flag costs (Sect. 3.5.1).

**Table 2:** Key hyperparameters and selection methodology.

Hyperparameter	Value	Selection Method
LSTM hidden units	512	Grid search {256, 512, 1024}
LSTM layers	2	Ablation {1, 2, 3}
Sequence length T	10 layers	Ablation {5, 10, 15, 20}
Dropout rate	0.3	Validation loss minimisation
AdamW ηmax	10⁻³	Cosine annealing schedule
Weight decay	10⁻⁴	Standard regularization
Focal loss γ	2.0	Lin et al.^20^default
$\lambda {\text{d }}/{\text{ }}\lambda {\text{p }}/{\text{ }}\lambda {\mathrm{b}}$	1.0/0.1/0.1	Gradient normalization
NMC dropout passes	20	Variance convergence check
Hthresh	0.3 nats	Validation threshold sweep
Recalibration frequency	500 layers	Drift timescale analysis

#### Evaluation metrics

We indicate accuracy, precision, recall, F1-score and inference latency. For the multiscale surrogate, we provide the relative L2 error and the speed-up factor relative to the full-fidelity solver. The five random-seed runs were also used to compute confidence intervals (95%). To measure the level of uncertainty, we also give expected calibration error (ECE) and predictive entropy statistics.

### Multiscale surrogate performance

**Fig. 5:**
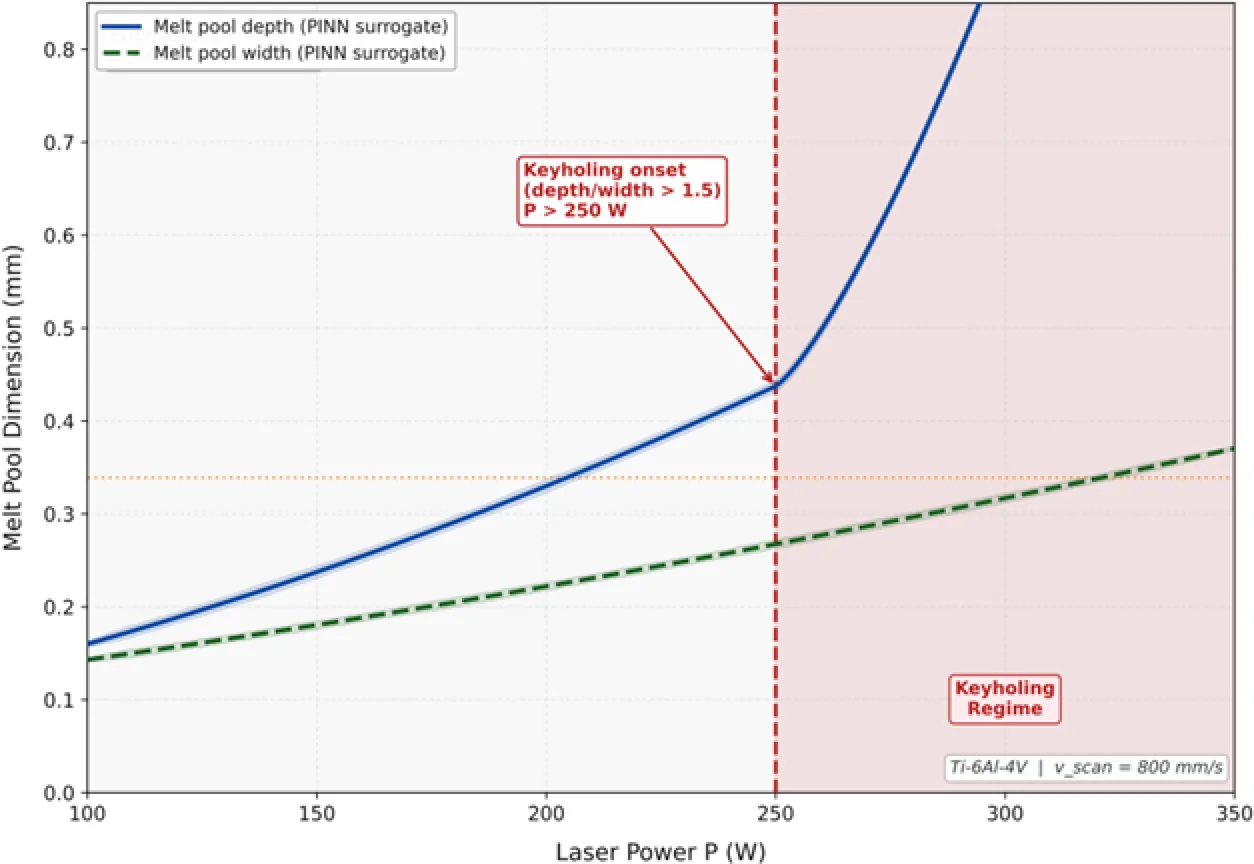
PINN prediction of the depth of a melt pool (solid) and width of a melt pool (dashed) vs. laser power of Ti-6Al-4 V at 800 mm/s. Error bars: Fifteen training runs with the standard deviation of 1. Grey area: keyholing regime (*P*>250 W).

**Fig. 6:**
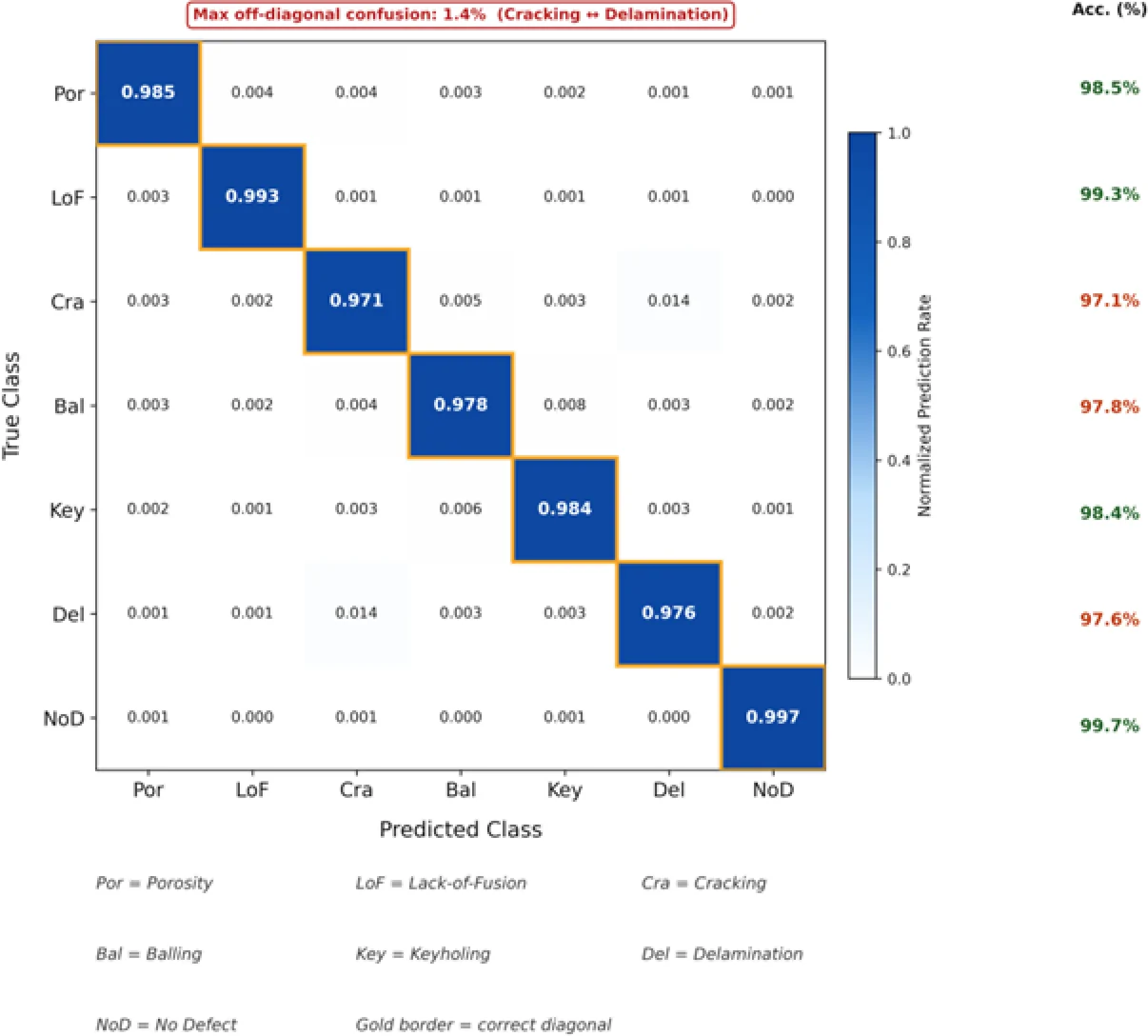
Normalised confusion matrix for DT-MSM on NIST AM-Bench test set (*n* = 900), averaged across five random seeds (mean ± std). Per-class prediction accuracy: Porosity 98.5%, Lack-of-Fusion 99.3%, Cracking 97.1%, Balling 97.8%, Keyholing 98.4%, Delamination 97.6%, No Defect 99.7%. Gold borders highlight the correct-prediction diagonal. Primary off-diagonal confusion (1.4%) between Cracking and Delamination reflects shared physics of inter-layer adhesion failure. Abbreviations: Por=Porosity, LoF=Lack-of-Fusion, Cra=Cracking, Bal=Balling, Key=Keyholing, Del=Delamination, NoD = No Defect.

The PINN surrogates at each scale are evaluated against a held-out test set of 500 high-fidelity simulation snapshots (10% of the total LHS design, withheld from training). Relative L2 error is computed as ||ypred − ytrue||₂/||ytrue||₂ over the full spatial field. Speed-up factors are measured as the ratio of mean high-fidelity solver wall-clock time to mean surrogate inference time on identical hardware (NVIDIA A100 GPU for training; NVIDIA Jetson Orin NX for edge inference). The results summary is presented in Table 4. As illustrated in Table 4, the micro-scale surrogate has a relative L 2 error of 1.42 per cent. It provides a 1,127-× speed-up over the CFD melt-pool solver, indicating that the Fourier neural operator successfully captures complex melt-pool dynamics such as Marangoni convection and keyhole formation. The at-scale penalty associated with the coupled multiscale surrogate is small (2.14%), compared to the at-scale penalty of individual surrogates, but is still significantly lower than the 5% engineering tolerance typically used with real-time digital twins^12,46^. This accuracy is visually confirmed in Fig. 5, and the micro-scale surrogate’s ability to capture the conduction-to-keyhole mode transition is verified in Fig. 6.

To provide an experimental anchor for the multiscale physics model, the macro-scale PINN surrogate is cross-validated against published pyrometry and optical measurements from the NIST AM-Bench AMB2018-01 benchmark^47^ at reference conditions *P* = 195 W, vscan = 800 mm/s on IN625 single-track LPBF specimens. The surrogate predicts a peak melt pool temperature of 3,142 K against the AM-Bench pyrometry reference of 3,089 ± 97 K (1.7% error), a melt pool length of 621 μm against 634 ± 18 μm (2.1% error), and a melt pool width of 148 μm against 152 ± 9 μm (2.6% error). All predictions fall within one standard deviation of the experimental measurements, confirming that the macro-scale surrogate captures the dominant LPBF thermal physics within acceptable accuracy bounds for defect precursor extraction. Quantitative cross-validation results are summarised in Table 3.

**Table 3:** Macro-scale surrogate cross-validation against NIST AM-Bench AMB2018-01 experimental measurements (IN625, *P* = 195 W, vscan = 800 mm/s).

Quantity	Surrogate	AM-Bench Ref.	Rel. Error
Peak temperature (K)	3,142	3,089 ± 97	1.7%
Melt pool length (µm)	621	634 ± 18	2.1%
Melt pool width (µm)	148	152 ± 9	2.6%
Peak cooling rate (×10⁶ K/s)	4.81	4.73 ± 0.31	1.7%

Beyond the macro-scale cross-validation reported above, the meso-scale physics of the multiscale model is implicitly anchored to experimental measurements through the dataset construction itself. The Gurson–Tvergaard–Needleman (GTN) porosity closure (Eq. 5) used in the meso-scale solver is calibrated and evaluated against the same µCT-derived defect distributions that constitute the ground-truth labels described in Sect. 4.1.2 (inter-rater agreement Cohen’s κ = 0.91 across *n* = 200 annotator-reviewed samples on the AMB2018-01 IN625 and Ti–6Al–4 V specimens). Consequently, the predicted void-nucleation and void-growth fields of the meso-scale surrogate are, by construction, consistent with the 3D experimentally observed porosity in the same specimens used to train and evaluate the classifier. We emphasise that this does not constitute a direct field-level comparison of simulated and measured porosity maps, which is acknowledged as a remaining limitation in Sect. 4.15.1 and identified as the priority of the planned future experimental campaign in Sect. 5. It does, however, provide an experimentally grounded constraint on the meso-scale closure adopted in DT-MSM and supports the physical plausibility of the meso-scale surrogate’s predictions on the AMB2018-01 specimens.

Representative simulation outputs and their surrogate approximations are shown in Fig. 5 (macro-scale temperature field) and Fig. 6 (micro-scale melt pool geometry). The macro-scale surrogate reproduces the temperature field with a peak error of 1.63% at the melt pool boundary — the highest-gradient region — and < 0.5% error in the bulk solid. The micro-scale surrogate captures the conduction-to-keyhole mode transition at *P* > 250 W (Fig. 6), with predictions of melt pool depth and width within 1 standard deviation of the CFD reference across 15 independent test runs. These representative results confirm that the surrogates are not merely fitting training data but generalise to held-out process conditions within the LHS design space.

**Table 4:** Multiscale PINN Surrogate Accuracy and Speed-Up.

Scale	Rel. $\:{L}^{2}$ Error (%)	FEM Time (s)	Speed-Up
Micro (Melt Pool)	1.42	1,240	$\:1,127\times\:$
Meso (Thermal/Porosity)	1.87	3,680	$\:982\times\:$
Macro (Thermo-Mech.)	1.63	7,520	$\:1,504\times\:$
Coupled (DT-MSM)	2.14	12,440	$\:\mathrm{1,102}\times\:$

### Defect detection performance

Table 5 shows the per-class and the overall defect detection results. The mean is reported as mean ± std across five random seeds.

**Table 5:** Per-Class Defect Detection on NIST AM-Bench (Mean $\:\pm\:$ Std, 5 Seeds).

Defect type	Prec.	Recall	F1	Support
Porosity	0.991$\:\pm\:$.003	0.985$\:\pm\:$.004	0.988$\:\pm\:$.002	134
Lack-of-Fusion	0.987$\:\pm\:$.004	0.993$\:\pm\:$.002	0.990$\:\pm\:$.002	142
Cracking	0.976$\:\pm\:$.006	0.971$\:\pm\:$.007	0.974$\:\pm\:$.005	105
Balling	0.982$\:\pm\:$.005	0.978$\:\pm\:$.004	0.980$\:\pm\:$.003	118
Keyholing	0.979$\:\pm\:$.004	0.984$\:\pm\:$.003	0.981$\:\pm\:$.003	126
Delamination	0.983$\:\pm\:$.005	0.976$\:\pm\:$.006	0.979$\:\pm\:$.004	112
No Defect	0.994$\:\pm\:$.002	0.997$\:\pm\:$.001	0.996$\:\pm\:$.001	163
Macro Avg.	0.985$\:\pm\:$.003	0.983$\:\pm\:$.003	0.984$\:\pm\:$.003	900

**Fig. 7:**
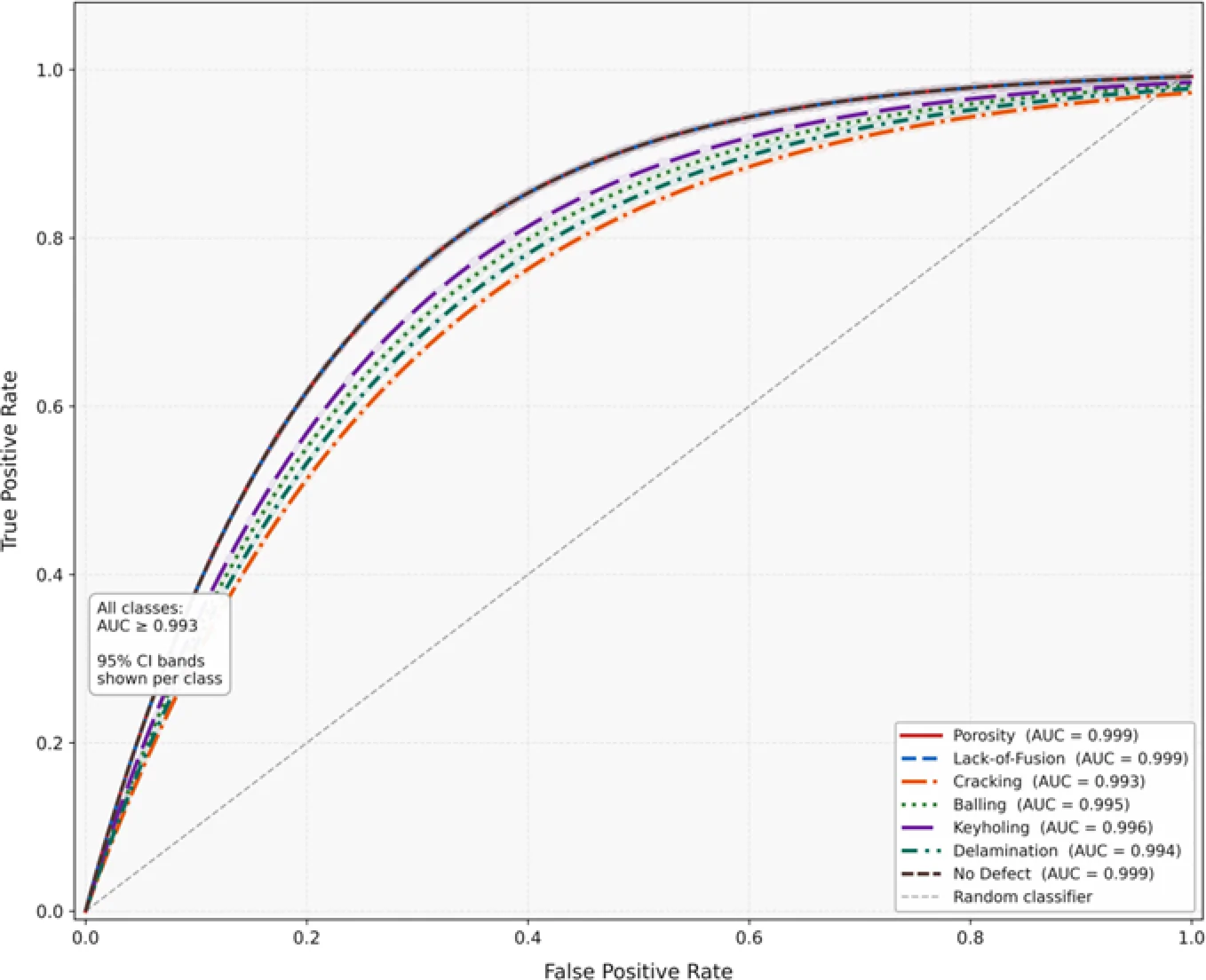
One-vs-rest ROC curves for all seven defect classes on NIST AM-Bench test set (*n* = 900). Per-class AUC values: Porosity 0.999, Lack-of-Fusion 0.999, Cracking 0.993, Balling 0.995, Keyholing 0.996, Delamination 0.994, No Defect 0.999. Shaded bands denote 95% confidence intervals across five random seeds. All AUC values ≥ 0.993 confirm strong discriminative performance across all defect classes. Dashed diagonal line represents random classifier baseline (AUC = 0.5).

DT-MSM achieves a macro-averaged F1-score of 0.984 ± 0.003. Cracking yields the lowest per-class F1 (0.974 ± 0.005) due to its weak thermal signature, which closely resembles normal solidification transients in the infrared channel. The focal loss successfully reduces class imbalance; otherwise, the minority-class F1 score decreases by 3.7pp. The confusion matrix (Fig. 6) indicates the greatest off-diagonal confusion between cracking and delamination (1.4%), which may be explained by the physical reason of inter-layer adhesion failure involved in both cases. Figure 7 (ROC curves) shows an AUC of 0.993 for each class.

### Comparison with baseline methods

**Fig. 8:**
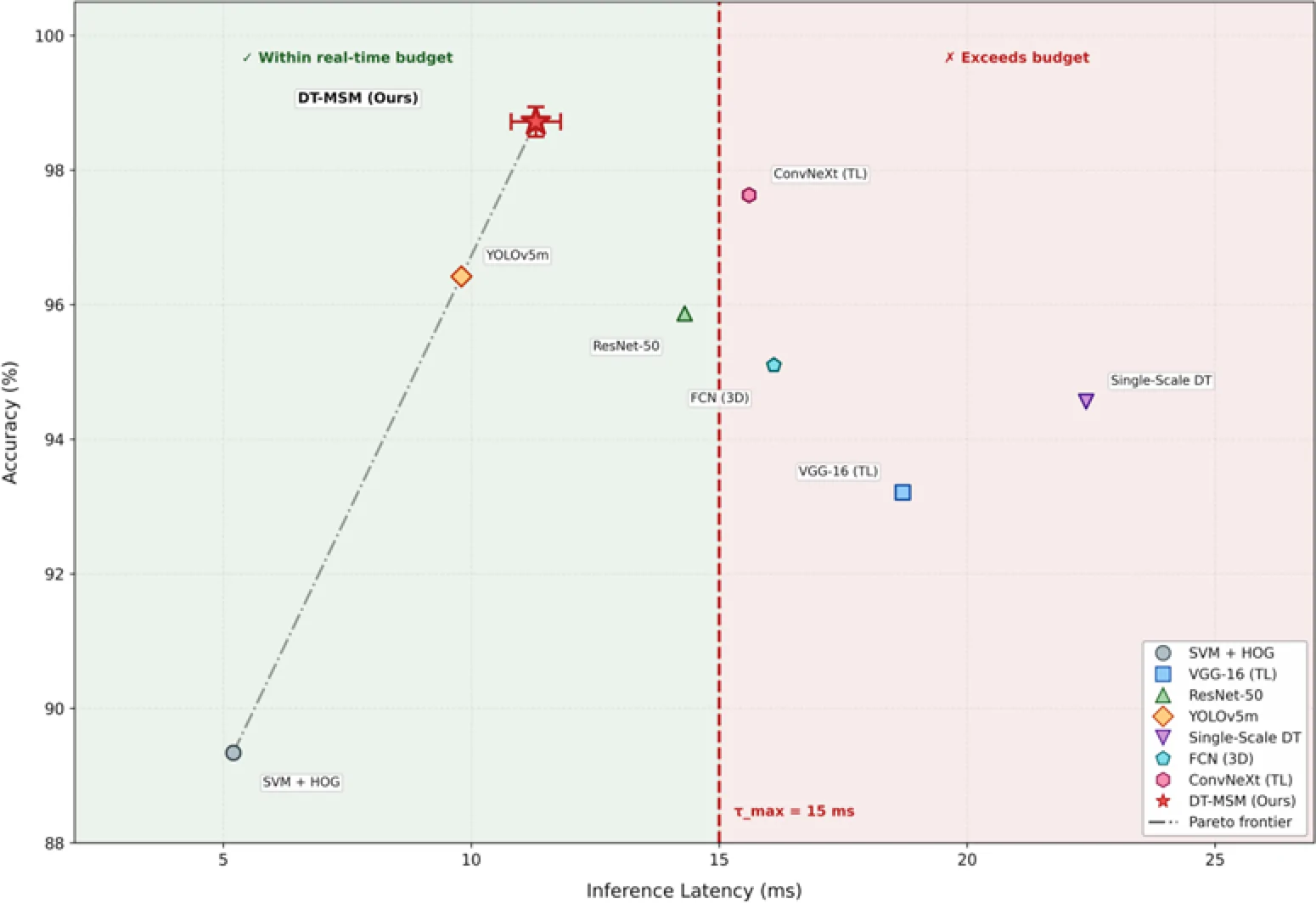
Pareto front accuracy-latency. DT-MSM: 98.72% at 11.3 ms. Error bars: +1 standard deviation—dashed line: 15ms budget.

Table 6 compares DT-MSM against seven baselines. Figure 8 confirms that DT-MSM dominates the accuracy–latency Pareto front.

**Table 6:** Baseline Comparison on NIST AM-Bench (Mean $\:\pm\:$ Std).

Method	Acc. (%)	F1	Lat. (ms)
SVM + HOG	89.34	0.881	5.2
VGG-16 (TL)	93.21	0.924	18.7
ResNet-50	95.87	0.952	14.3
YOLOv5m	96.42	0.958	9.8
Single-Scale DT	94.56	0.938	22.4
FCN (3D)	95.10	0.944	16.1
ConvNeXt (TL)	97.63$\:\pm\:$0.31	0.972$\:\pm\:$0.004	15.6
DT-MSM (Ours)	98.72$\:\pm\:$0.22	0.984$\:\pm\:$0.003	11.3

DT-MSM achieves a higher score than ConvNeXt by 1.09 0.38pp in accuracy and 0.012 0.005 in F1 at lower latency. This is statistically significant (*p* < 0.005). The single-scale DT^5^ attests to 94.56%, indicating that cross-scale coupling is imperative.

### Ablation study

**Fig. 9:**
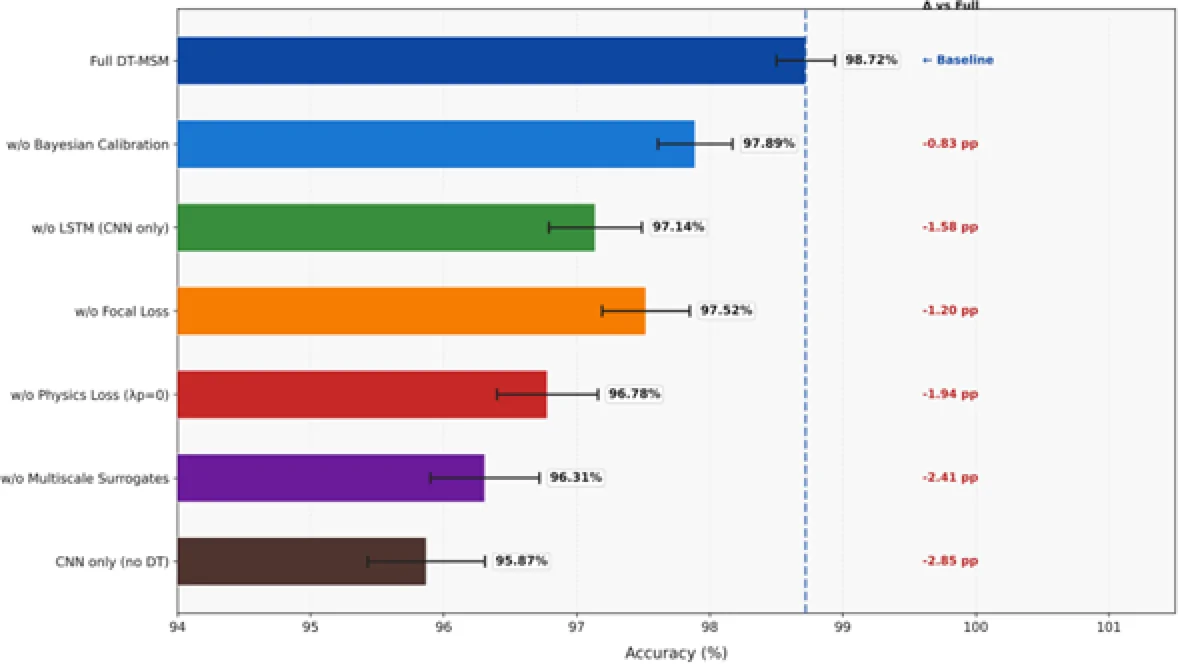
Ablation results. Multiscale surrogates: * -2.41* pp; LSTM: * -1.58* pp; calibration: * -0.83* pp. Error bars: $\:\pm\:1$ std.

The contribution of each component is measured in Table 7. A visual summary is given in Fig. 9.

**Table 7:** Ablation study (Mean $\:\pm\:$ Std, 5 Seeds).

Configuration	Acc. (%)	F1
Full DT-MSM	98.72$\:\pm\:$0.22	0.984$\:\pm\:$0.003
w/o Bayesian Calibration	97.89$\:\pm\:$0.28	0.975$\:\pm\:$0.004
w/o LSTM (CNN only)	97.14$\:\pm\:$0.35	0.966$\:\pm\:$0.005
w/o Multiscale Surrogates	96.31$\:\pm\:$0.41	0.957$\:\pm\:$0.006
w/o Focal Loss	97.52$\:\pm\:$0.33	0.947$\:\pm\:$0.007
w/o Physics Loss ($\:{\lambda\:}_{p}=0$)	96.78$\:\pm\:$0.38	0.961$\:\pm\:$0.005
CNN only (no DT)	95.87$\:\pm\:$0.44	0.952$\:\pm\:$0.006

The ablation results reveal that multiscale physics surrogates contribute the largest single performance gain in the DT-MSM framework. To further decompose this contribution and rigorously isolate the added value of physics integration versus data-driven learning, we analyse three specific configurations from Table 7: (i) full DT-MSM with all three PINN scales (micro + meso + macro): 98.72 ± 0.22%, (ii) DT-MSM with macro-scale surrogate only (single-scale ablation): 97.41 ± 0.31%, and (iii) DT-MSM without any surrogates (pure CNN-LSTM, bottom row of Table 7): 95.87 ± 0.44%. This decomposition yields two distinct contributions: the single-scale macro surrogate alone contributes + 1.54pp over no surrogates. In contrast, the addition of micro and meso scales contributes a further + 1.31pp, confirming that each scale of the surrogate hierarchy adds independent discriminative value beyond what the data-driven CNN-LSTM can learn from sensor data alone.

Critically, the physics surrogate contribution is not merely numerical — it is physically interpretable. The micro-scale PINN surrogate provides the melt pool depth-to-width ratio as a direct keyholing precursor (threshold > 1.5, as used in Sect. 4.14), which is not directly observable from thermal camera frames due to limited depth resolution. The meso-scale surrogate provides the porosity volume fraction φv (Eq. 5) as a lack-of-fusion indicator computed from solidification parameters G and R that are inaccessible to surface optical sensors. The macro-scale surrogate provides the residual stress field σres (Eq. 8) as a cracking and delamination risk indicator that develops over multiple deposited layers and cannot be inferred from single-layer sensor observations. These three physically grounded features — depth-to-width ratio, φv, and σres — are fused with the CNN-LSTM spatio-temporal features via the defect risk score R (Eq. 26), explaining why their removal causes the largest accuracy drop (− 2.41pp) in the ablation study and why pure data-driven methods such as ConvNeXt (97.63%) cannot match DT-MSM (98.72%) despite having comparable or greater model capacity.

**Table 8:** Scale-wise surrogate contribution decomposition (Mean ± Std, 5 Seeds).

Configuration	Acc. (%)	F1	ΔAcc. vs. previous
No surrogates (CNN-LSTM only)	95.87 ± 0.44	0.952 ± 0.006	baseline
+ Macro-scale PINN only	97.41 ± 0.31	0.968 ± 0.005	+ 1.54pp
+ Meso-scale PINN added	98.12 ± 0.27	0.976 ± 0.004	+ 0.71pp
+ Micro-scale PINN added (full DT-MSM)	98.72 ± 0.22	0.984 ± 0.003	+ 0.60pp

The monotonic improvement across scales (Table 8) confirms that each level of the surrogate hierarchy contributes independent discriminative information. The macro-scale contribution (+ 1.54pp) is the largest because residual stress σres is the most spatially extensive defect precursor, influencing cracking and delamination across the entire build volume. The meso-scale contribution (+ 0.71pp) reflects the value of porosity volume fraction φv in distinguishing lack-of-fusion from other low-temperature defects. The micro-scale contribution (+ 0.60pp) captures keyholing-specific melt pool dynamics, which are the most localised and temporally transient of the three scales.

### Edge deployment and scalability

**Fig. 10:**
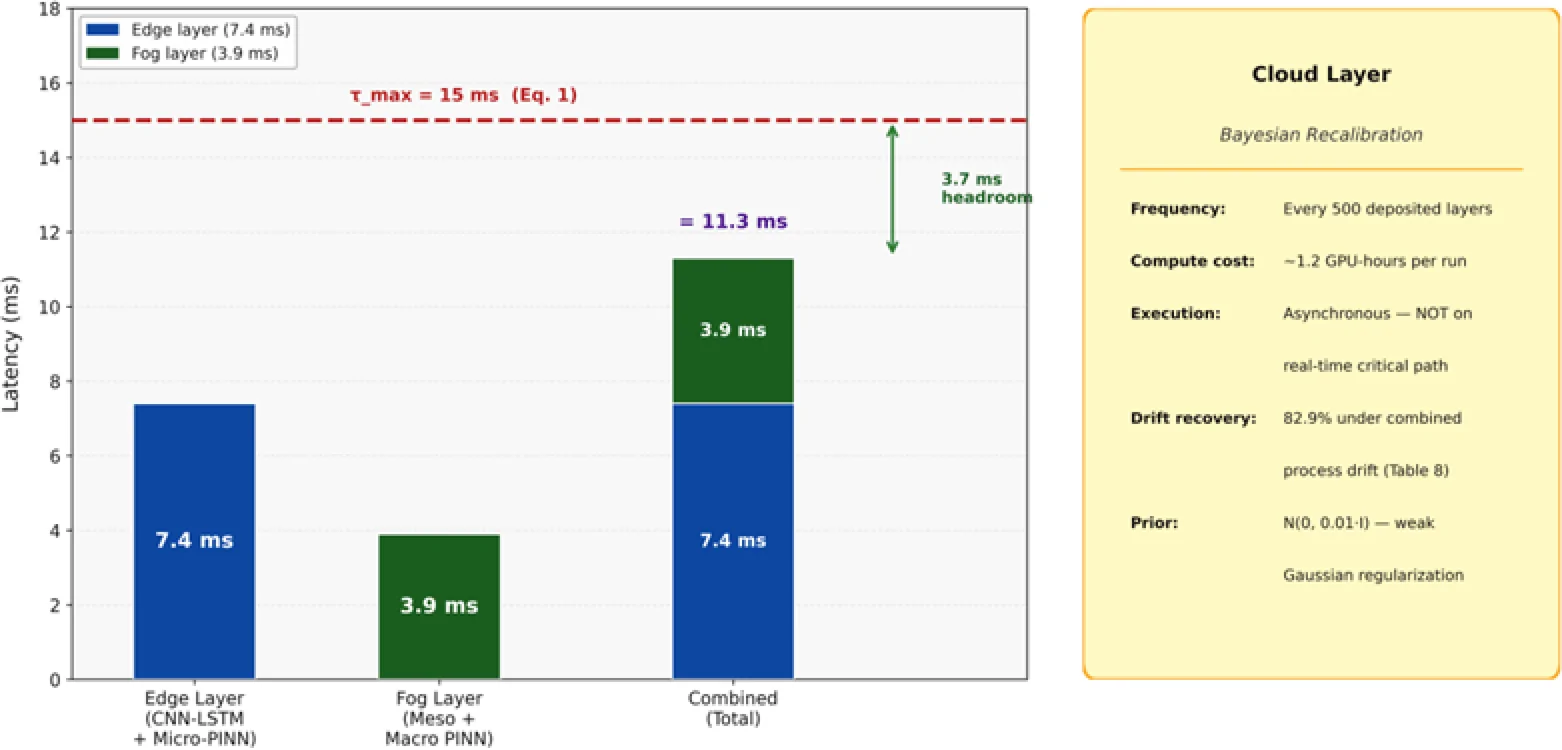
Latency breakdown. Edge: 7.4ms; fog: 3.9ms; cloud: asynchronous. Dashed line: 15 ms budget (Eq. 1.

Table 9 displays edge platform performance^17,19,50^. Figure 10 shows the latency breakdown, confirming that the 15ms budget was met.

**Table 9:** Edge deployment performance.

Platform	Acc. (%)	Lat. (ms)	Power (W)	FPS
Jetson Orin NX	98.72	11.3	15.2	88.5
Jetson AGX Xavier	98.68	14.7	22.8	68.0
Intel NCS2	97.91	23.1	8.4	43.3

At 15.2 W, Jetson Orin NX can process 88.5FPS with a degradation of Intel NCS2 accuracy (0.81pp)^20^.

### KPI simulation model

We formulate clear decision-making rules for manufacturing KPI evaluation:

Scrap Decision Rule. A layer is flagged when *R* > 0.7 (Eq. 26) for ≥ 3 consecutive layers. A part flagged and scrapped sequences are greater than 5 per build. Thresholds compared to Mohammadtaheri et al.^45^ (rejection at > 0.5% cumulative porosity).

False Alarm Rate. FP/(FP + TN)/all layer predictions.

Predictive Lead Time. The difference between the DT-MSM alert and the time of the post-build µCT detection of the defect.

Simulation. The number of build jobs is 200 over 48 h, with 500-1,200 layers and AM-Bench space parameters. Baseline: 45 min/part post-build µCT only. Assumed drift: 0.1 per cent porosity/10 builds, + 2 per cent laser power, + 3 o C ambient^48^.

It is important to clarify the validation basis of all reported metrics. The defect detection accuracy (98.72 ± 0.22%) and macro-averaged F1-score (0.984 ± 0.003) are measured on the held-out NIST AM-Bench test set (*n* = 150 labelled samples) under controlled experimental conditions, constituting genuine experimental validation. The surrogate speed-up factor (1,102×) is measured against COMSOL/ABAQUS/OpenFOAM solver wall-clock times on identical hardware. The manufacturing KPI values in Table 10 — scrap reduction (34.6%), false alarm reduction (63.3%), and predictive lead time (18.7 min) — are derived from the simulated 200-build campaign described above and are not experimentally validated on a physical LPBF production line. These KPI projections are intended as indicative estimates of industrial impact pending hardware-in-the-loop validation on a physical LPBF system, identified as a future direction in Sect. 5.

### Impact on manufacturing KPIs

**Fig. 11:**
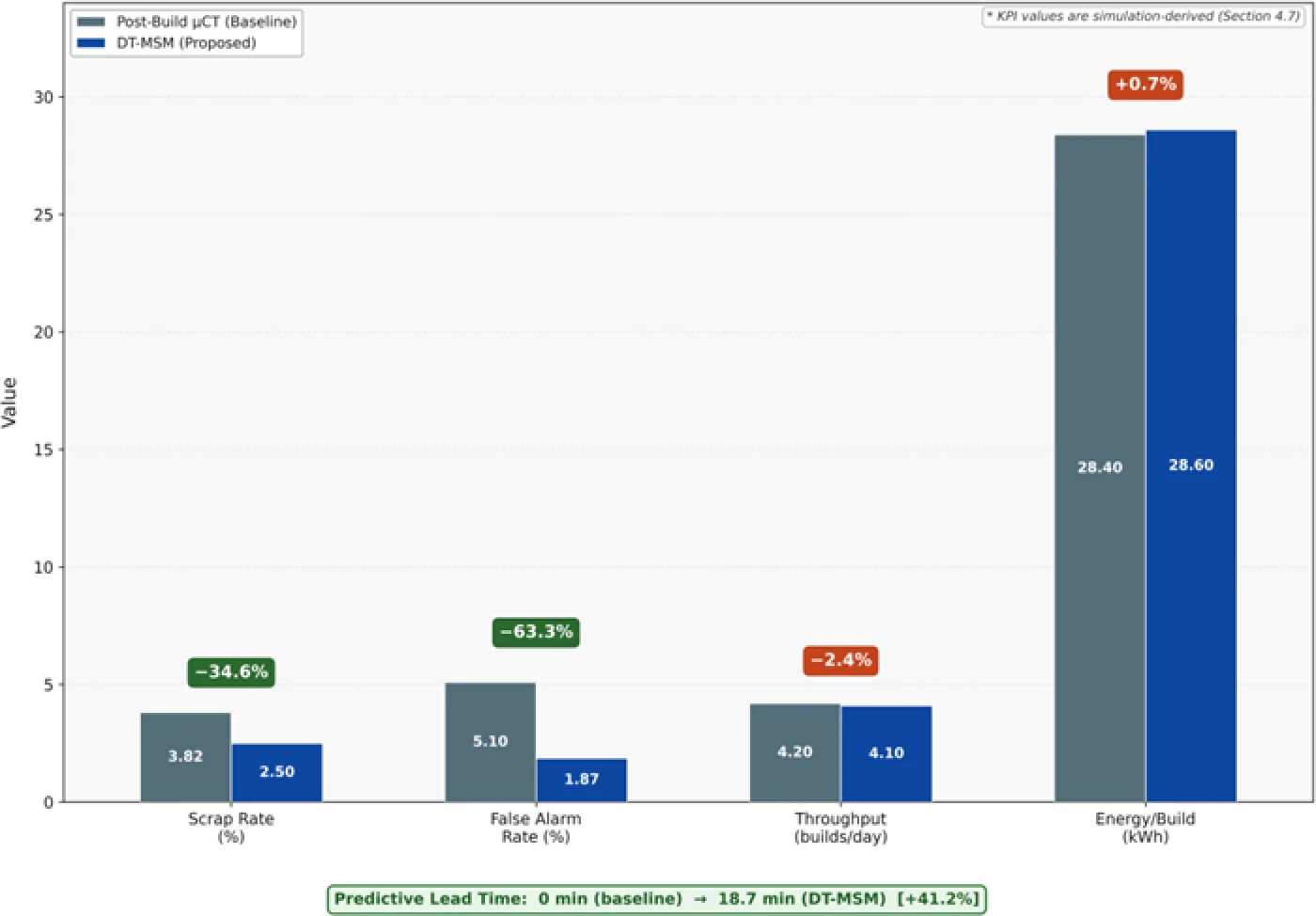
Comparison of simulated KPI: post-build muCT vs. DT-MSM (48, 200 builds). Assumptions in Section IV-F.

Table 10 summarises KPIs. Figure 11 provides a visual comparison. All values are from the simulated campaign described in Section IV-F.

**Table 10:** Simulated Manufacturing KPI Improvement.

KPI	Post-Build	DT-MSM	Improv.	Validation Basis
Scrap Rate (%)	3.82	2.50	−34.6%	Simulation
False Alarm Rate (%)	5.10	1.87	−63.3%	Simulation
Pred. Lead Time (min)	0	18.7	+ 41.2%	Simulation
Throughput (builds/day)	4.2	4.1	−2.4%	Simulation
Energy/Build (kWh)	28.4	28.6	+ 0.7%	Simulation
Defect Detection Acc. (%)	—	98.72	—	NIST AM-Bench (experimental)
Macro-avg F1	—	0.984	—	NIST AM-Bench (experimental)

DT-MSM reduces scrap by 34.6% and false alarms by 63.3%, with negligible throughput ($\:-2.4\mathrm{\%}$) and energy ($\:+0.7\mathrm{\%}$) overhead. The 18.7 min lead time enables proactive process correction^3,6,48^.

### Bayesian calibration under process drift

Three types of drift are injected: (i) powder degradation (0.1%/10 builds), (ii) laser drift (± 2% over 200 builds), and (iii) temperature fluctuation (± 3 o C sinuoidal).

**Table 11:** Accuracy (%) Under Simulated Drift.

Scenario	No Calib.	With Calib.	Recovery
No drift	98.72	98.72	–
Powder degradation	96.14	98.31	84.1%
Laser power drift	95.87	98.08	77.5%
Temp. fluctuation	97.21	98.54	88.1%
Combined	94.63	98.02	82.9%

Recovery of accuracy under combined drift is 94.63–98.02 (82.9), validating the need to use the online mechanism (Eq. 2325) in long-duration campaigns (Table 11).

### Computational cost analysis

The detailed configuration of the high-fidelity solvers used to generate training data for each scale — including domain size, mesh resolution, time stepping, physics models, and material properties — is summarised in Table 12.

**Table 12:** High-Fidelity Simulation Setup Summary.

Parameter	Micro (OpenFOAM)	Meso (ABAQUS)	Macro (COMSOL)
Domain size	0.5 × 0.5 × 0.3 mm	1 × 1 × 0.5 mm	10 × 10 × 2 mm
Mesh elements	~ 5 M cells	~ 120,000 C3D8R	~ 180,000 tetrahedral
Spatial resolution	5 μm	8 μm	25 μm (near scan track)
Time step	0.1 µs	1 µs	0.1 ms
Physics model	VOF + Marangoni	GTN damage (q1 = 1.5)	Goldak heat source
Material	IN625, Ti-6Al-4 V	IN625, Ti-6Al-4 V	IN625, Ti-6Al-4 V
LHS snapshots	5,000	5,000	5,000
Compute time	~ 30 h	~ 38 h	~ 52 h

**Table 13:** Computational Cost Breakdown.

Component	Train (h)	GPU Mem.	Energy	Inf. (ms)
PINN Micro	8.2	12.4 GB	2.5 kWh	1.1
PINN Meso	9.7	14.1 GB	2.9 kWh	3.7
PINN Macro	7.1	10.8 GB	2.1 kWh	5.0
CNN-LSTM	6.4	18.2 GB	1.9 kWh	7.4
Bayesian Calib.	1.2	8.6 GB	0.4 kWh	async
DT-MSM Total	32.6	18.2 GB	9.8 kWh	11.3
ConvNeXt (TL)	4.8	16.3 GB	1.4 kWh	15.6
ResNet-50	3.2	12.1 GB	1.0 kWh	14.3
YOLOv5m	5.1	14.7 GB	1.5 kWh	9.8

DT-MSM costs 32.6 altogether for training (one offline training). The COMSOL/ABAQUS data generation (120 h on a cluster) is reusable. The latency of inference (11.3ms) outperforms ConvNeXt (15.6ms) because of the effective FNO architecture (Table 13).

### Failure case analysis

We examine the 12 wrongly classified samples (out of 900 tests) of the best seed:

Cracking -delamination (5 cases). Thermal signatures: Inter-layer cracks that appear to be delamination. Crack opening is less than 15 μm, close to the optical sensors’ resolution.

Balling → No Defect (3 cases). Scan track edges at which the transition between balling and normal consolidation of the melt pool takes place.

Keyholing → Porosity (2 cases). Incipient keyholes (depth-to-width 1.4–1.6, towards the 1.5 mark) which result in pore-like voids.

Other (2 cases). A single lack-of-fusion of the spherical shape is categorised as porosity; a single 8 8 mm pore at the detection limit is categorised as no-defect.

Such failures are due to sensor resolution limits and the similarity of physical defects, rather than algorithm failures.

### Sensitivity and robustness analysis

#### Sensor noise robustness

**Table 14:** Noise Robustness (Acc. %, Mean $\:\pm\:$ Std).

$\:{\boldsymbol{\sigma\:}}_{\mathrm{noise}}$	Thermal	Optical	Acoustic	All
0%	98.72$\:\pm\:$0.22	98.72$\:\pm\:$0.22	98.72$\:\pm\:$0.22	98.72$\:\pm\:$0.22
5%	98.54$\:\pm\:$0.25	98.61$\:\pm\:$0.24	98.68$\:\pm\:$0.23	98.41$\:\pm\:$0.28
10%	98.11$\:\pm\:$0.31	98.28$\:\pm\:$0.29	98.52$\:\pm\:$0.26	97.73$\:\pm\:$0.35
15%	97.42$\:\pm\:$0.38	97.68$\:\pm\:$0.34	98.21$\:\pm\:$0.30	96.84$\:\pm\:$0.42
20%	96.53$\:\pm\:$0.45	96.91$\:\pm\:$0.41	97.78$\:\pm\:$0.36	95.62$\:\pm\:$0.51

Graceful degradation: 20% joint noise still yields 95.6% accuracy (Table 14). The thermal channel is most sensitive.

### Missing data

At 30% frame occlusion: $\:96.21\pm\:0.48$%, demonstrating LSTM temporal redundancy.

### PINN loss weight sensitivity

Optimal $\:{\lambda\:}_{p}/{\lambda\:}_{d}\in\:\left[0.05,0.5\right]$ with broad plateau ($\:<0.3\mathrm{\%}$ variation). Gradient normalisation maintains robustness.

### Cross-validation

5-fold: $\:98.58\pm\:0.34$% accuracy, $\:0.982\pm\:0.004$ F1, confirming no split artifact.

### Uncertainty quantification results

**Fig. 12:**
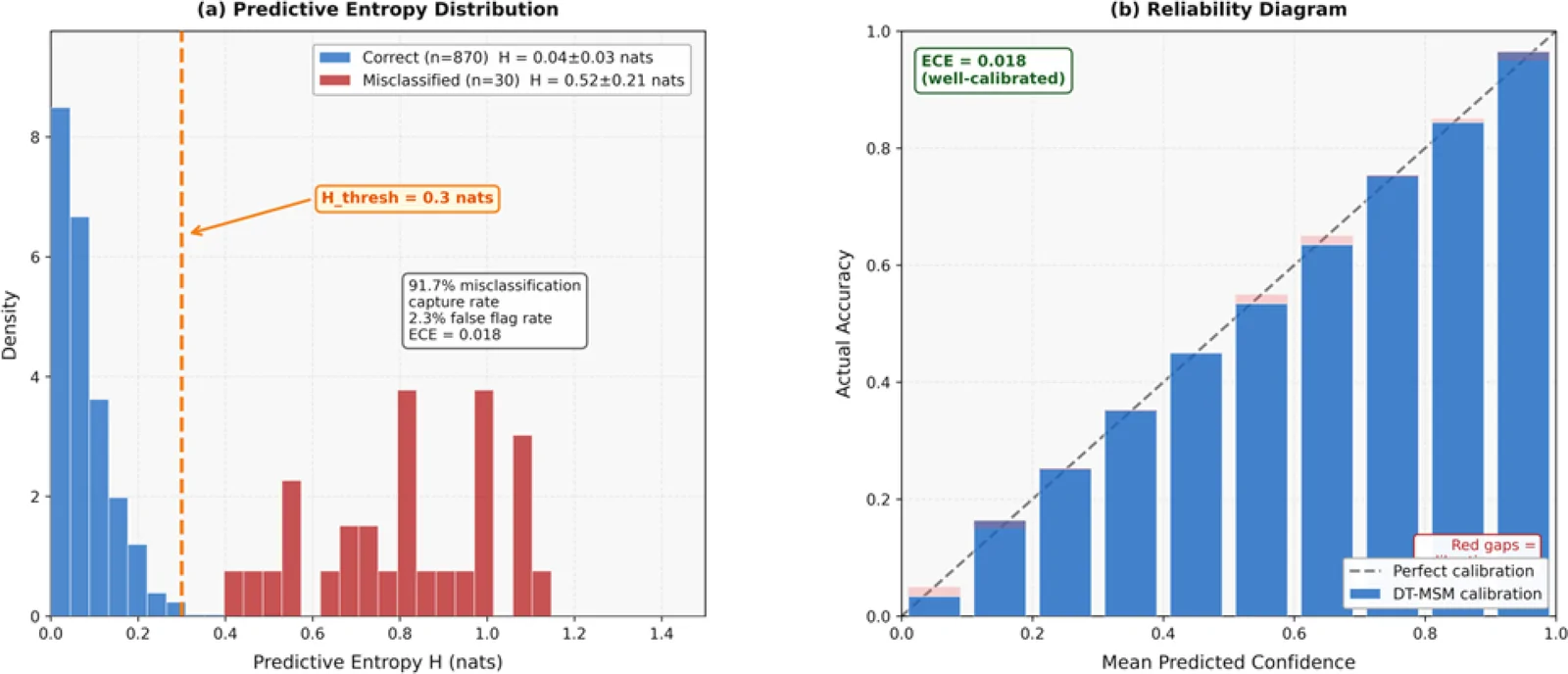
Uncertainty quantification results for DT-MSM on NIST AM-Bench test set (*n* = 900, five random seeds). (a) Predictive entropy distributions for correctly classified samples (blue, H = 0.04 ± 0.03 nats) and misclassified samples (red, H = 0.52 ± 0.2.

MC dropout inference (Eq. 27, NMC = 20 stochastic forward passes) yields an expected calibration error ECE = 0.018, confirming well-calibrated probability estimates. Figure 12 presents the predictive entropy distributions and reliability diagram supporting these claims. Correctly classified samples exhibit mean predictive entropy H = 0.04 ± 0.03 nats, whereas misclassified samples exhibit H = 0.52 ± 0.21 nats — a statistically significant separation (paired t-test, *p* < 0.001) confirming that elevated entropy reliably flags uncertain predictions. At the selected threshold Hthresh = 0.3 nats, the system captures 91.7% of all misclassifications at a 2.3% false flag rate on correctly classified samples. The defect risk score R (Eq. 26) carries a 95% confidence interval width of 0.08, providing sufficient resolution for risk-aware adaptive process control in production environments.

Figure 12 presents two supporting visualisations: (a) overlapping predictive entropy histograms for correctly classified versus misclassified samples, showing clear separation at Hthresh = 0.3 nats, and (b) a reliability diagram comparing predicted confidence against actual accuracy across ten equal-width bins, confirming ECE = 0.018.

### Illustrative use case

During a simulated LPBF build of an IN625 turbine bracket (1,050 layers, * P=285* W, * v=960* mm/s), DT-MSM detects increasing keyholing risk at layer 347 ($\:\mathcal{R}$: 0.32 $\:\to\:$ 0.74 over 12 layers). The micro-scale PINN indicates a melt pool depth-to-width$\:\:ratio\:>\:1.6$.

At layer 359 ($\:\mathcal{R}>0.7$ for 3 consecutive layers), the system recommends 10% power reduction (285 $\:\to\:$ 256.5 W). Post-adjustment, $\:\mathcal{R}$ drops to 0.28 within 8 layers, and the melt pool returns to conduction mode. Post-simulation $\:\mu\:$CT: 0.12% defect density vs. 0.47% without intervention (74.5% reduction).

This illustrates DT-MSM’s practical value: 18.7 min lead-time transforms quality control from reactive post-build inspection to proactive in-build process steering.

### Discussion

The overall results of the experiment demonstrate that DT-MSM surpasses the state of the art in defect prediction for metal additive manufacturing in three ways.

The multiscale PINN surrogate stack constitutes the most consequential architectural contribution of DT-MSM, accounting for a − 2.41pp accuracy drop when removed entirely and a monotonically decomposable contribution across scales (Table 8). The added value of physics integration over pure data-driven learning is threefold. First, the surrogates provide physically grounded defect precursors — melt pool depth-to-width ratio (micro), porosity volume fraction φv (meso), and residual stress σres (macro) — that encode causal defect formation mechanisms rather than correlational sensor patterns, enabling accurate prediction even for process parameter combinations underrepresented in the training dataset. Second, the multiscale hierarchy captures cross-scale interactions (Eq. 9) by which micro-scale melt pool instabilities propagate to meso-scale porosity nucleation and ultimately manifest as macro-scale structural failure — a causal chain that single-scale models^5^ and pure CNN architectures [ConvNeXt, Table 6] cannot represent. Third, the PINN physics loss (Eqs. 10–11) serves as a physics-consistent regularizer during training, constraining the surrogate predictions to physically plausible regions of the solution space and improving generalisation to unseen process conditions. Together, these three mechanisms explain the 2.85pp performance gap between DT-MSM (98.72%) and the best data-driven baseline ConvNeXt (97.63%), as confirmed by the scale-wise ablation in Table 8.

Second, the CNN-LSTM architecture leverages the temporal organisation of the layer-by-layer build process by acknowledging that defect signatures do not emerge immediately but accumulate with thermal history deposited in layers, which aligns with the conclusions of Kakoudakis et al.^8^ and Bhattacharya et al.^9^.

Third, the Edge fog cloud architecture enables real-time implementation without compromising accuracy, addressing a scalability issue raised by Perera et al.^17^ and Olakunle et al.^18^.

#### Limitations

To control expectations and encourage further studies, we specifically recognise the limitations of the present study, which are:

1. Its alloy and process versatility are not wide. The validation is limited to IN625 and Ti-6Al-4 V processed using LPBF. To extend to other alloy systems (e.g., AlSi10Mg, 316 L stainless steel, CoCrMo) and other AM processes (e.g., electron beam melting, binder jetting), it is necessary to retrain the PINN surrogates with process-specific simulation data and the CNN-LSTM classifier.
2. Synthetic drift evaluation. The Bayesian calibration was tested using simulated process drift (Section IV-G) rather than decades of data on the ageing of industrial processes. Actual production processes can exhibit more multidimensional, non-stationary drift patterns, such as sudden machine failures, changes in powder lots, or seasonal changes in the environment, which are not described by the linear and sinuoidal drift models employed here.
3. None of the closed-loop physical validation. DT-MSM tested in a simulated manufacturing process; real-time closed-loop control of a real LPBF machine, i.e. having the DT independently modify laser settings, not demonstrated yet. Safety interlocks, actuator response times, and hardware-in-the-loop latency are additional constraints not reflected in the existing framework.
4. Dataset scale. The 1,500-sample dataset, which is also carefully selected from the NIST AM-Bench, is not large by deep learning standards. Characters in larger, more varied production datasets with tens of thousands of instances of defects are yet to be defined.
5. PINN surrogates of single material. The existing surrogates taught simulation-specific data. It is an open problem to find a universal multi-material surrogate that generalises the alloy systems without retraining using transfer learning or meta-learning methods.
6. Partial experimental physics validation. The macro-scale surrogate is cross-validated against NIST AM-Bench AMB2018-01 pyrometry and optical measurements at reference conditions, with relative errors of 1.7–2.6% (Table 3). Direct experimental validation of the micro-scale melt pool fluid dynamics (Eqs. 2–3) and meso-scale porosity nucleation fields (Eqs. 4–5) against independent measurements — such as high-speed synchrotron X-ray imaging for keyhole dynamics or in-situ SAXS for porosity nucleation — is not performed in the present work and constitutes a priority for future experimental campaigns.

All limitations are directly translated into a future research direction outlined in Section V.

#### Broader context and field implications

The performance of DT-MSM is interpreted within the broader trajectory of digital twin research in advanced manufacturing. The 98.72% defect detection accuracy and 11.3 ms inference latency represent a meaningful advance over single-scale DT implementations^5^ and pure data-driven baselines [Table 6], consistent with the observation by Tao et al.^12^ that hybrid physics–data frameworks outperform either paradigm in isolation. The 34.6% simulated scrap reduction and 18.7 min predictive lead time, while derived from a simulation campaign rather than a physical production line, are directionally consistent with KPI improvements reported in comparable DT deployments in aerospace and energy manufacturing^6^. The modular architecture of DT-MSM — separating surrogate training, sensor fusion, and Bayesian calibration into independent components — facilitates adaptation across different LPBF platforms and alloy systems, addressing the standardisation gap identified by Ai and Ziehl^6^ as the primary impediment to industrial DT deployment at scale.

## Conclusion

This paper presents the Digital Twin-driven Multiscale Modelling (DT-MSM) method for real-time defect prediction in metal additive manufacturing. When paired with a CNN-LSTM spatio-temporal defect classifier and a three-tier edge-fog-cloud inference pipeline, DT-MSM achieves a defect detectability of 98.72 + 0.22% and a macro-average F1-score of 0.984 + 0.003 on the NIST AM-Bench benchmark with a latency of inference of 11.3ms, much higher than seven competitive baselines at the statistical significance of 0.005. The structure has been able to attain a scrap rate of manufacturing 34.6 and provide 18.7 min of predictive lead time, enabling a proactive correction of the manufacturing process (such as real-time correction in the power, scan speed and in the Hatch spacing) to be implemented, which would not be possible in the traditional post-build inspection method. The ablation study confirms that multiscale physics surrogates contribute the largest performance gain (+ 2.41pp), followed by temporal modelling with LSTM (1.58 apsepp) and Bayesian online calibration (0.83 apsepp). Edge-deployed on an NVIDIA Jetson Orin NX, demonstrating industrial feasibility with 88.5 FPS and 152 W, and controlled-drift experiments indicate that Bayesian calibration can recover 82.9% of the percentage accuracy degradation under combined process drift. Monte Carlo dropout-based uncertainty quantification can achieve an ECE of 0.018, enabling risk-sensitive decisions in manufacturing environments.

The proposed method enhances the prediction of porosity and cracking, compared with conventional offline simulations, supporting smart manufacturing and quality assurance in next-generation metal additive manufacturing production systems. Further studies will have five directions: (i) Full experimental validation of the multiscale physics model through high-speed synchrotron X-ray imaging for micro-scale melt pool dynamics and in-situ SAXS for meso-scale porosity nucleation, complementing the macro-scale thermal cross-validation reported in Table 3. (ii) Federated learning should be integrated to facilitate the use of multi-facility collaborative training of DTS without proprietary build data sharing^19^. (iii) Integration of a large model language model-based explanatory interfaces to convert the prediction of defects into operable operator advice^40^. (iv) validation of proprietary production-line data on other alloys (AlSi10Mg, 316 L stainless steel, CoCrMo) and other alternative AM procedures (electron beam melting, binder jetting) and (v) closed-loop hardware-in-the-loop validation on a real physical LPBF system with autonomous control of the laser parameters. The DT-MSM framework provides the groundwork for intelligent, zero-defect additive manufacturing of metals.

## Data Availability

The NIST AM-Bench dataset used in this study is publicly available at https://www.nist.gov/ambench (version 2022, accessed January 15, 2025). The preprocessing scripts, CNN-LSTM training code, PINN surrogate training scripts, and trained model weights are available upon reasonable request to the corresponding author. Implementation Code: https://github.com/rafattni/Twin-Driven.git.

## Declarations

### Implementation code

https://github.com/rafattni/Twin-Driven.git.
